# A combination of differential expression and network connectivity analyses identifies a common set of RNA splicing and processing genes altered with age across human tissues

**DOI:** 10.18632/aging.206347

**Published:** 2025-12-22

**Authors:** Caio M.P.F. Batalha, André Fujita, Nadja C. de Souza-Pinto

**Affiliations:** 1 Department of Biochemistry, Chemistry Institute, University of São Paulo, São Paulo, São Paulo 05508-000, Brazil; 2 Department of Computer Science, Institute of Mathematics and Statistics, University of São Paulo, São Paulo, São Paulo 05508-000, Brazil; 3 Division of Network AI Statistics, Medical Institute of Bioregulation, Kyushu University, Fukuoka 812-8582, Japan

**Keywords:** aging, gene expression, co-expression network analysis, RNA processing

## Abstract

Although transcriptomic changes are known to occur with age, the extent to which these are conserved across tissues is unclear. Previous studies have identified little conservation in age-modulated genes in different tissues. Here, we sought to identify common transcriptional changes with age in humans (aged 20 to 70) across tissues using differential network analysis, assuming that differential expression analysis alone cannot detect all changes in the transcriptional landscape that occur in tissues with age. Our results demonstrate that differential connectivity analysis reveals significant transcriptional alterations that are not detected by differential expression analysis. Combining the two analyses, we identified gene sets modulated by age across all tissues that are highly enriched in terms related to “RNA splicing” and “RNA processing”. The identified genes are also highly interconnected in protein-protein interaction networks. Co-expression module analyses demonstrated that other genes that show tissue-specific variations with age are enriched in pathways that combat the accumulation of aberrant RNAs and proteins, likely caused by defective splicing. Additionally, with convergent connectivity patterns, most tissues significantly reorganized their gene connectivity with age. Our results identified genes and processes whose age-associated transcriptional changes are conserved across tissues, demonstrating a central role for RNA splicing and processing genes and highlighting the importance of differential network analysis for understanding the ageing transcriptome.

## INTRODUCTION

Aging is a multifactorial process that alters several essential pathways, increasing the risk of death. Despite its general nature, the underlying mechanisms leading to the aging phenotypes still need to be well established.

One pressing question in the field is whether common pathways are altered with age across different tissues. For instance, analyzing the methylation status of a small number of CpG sites (epigenetic clocks) allows for predicting an organism’s age with remarkable accuracy [1–5]. Notably, some of these epigenetic clocks are pan-tissue, developed using data generated from multiple tissues and representing a pattern of methylation changes common across several tissues [1, 3].

However, despite the success in identifying conserved DNA methylation patterns across tissues, finding common patterns with gene expression data has proven more challenging. Several studies have compared age-related changes in gene expression across tissues in humans [6–11] and rodents [6, 11, 12], with inconclusive results. While some report no overlap in differentially expressed genes with age between tissues, others find subtle commonalities between a few tissues at a process level but not at the gene level. Therefore, whether tissues age differently or if common aging alterations are reflected in conserved tissue-independent transcriptomic changes is unclear.

Gene expression profiles and biological outcomes result not only from variations in each gene’s specific expression levels but also from co-expression patterns. Gene co-expression is typically represented as a correlation that measures how coordinated the expression of any two genes is. Gene expression levels and gene co-expression patterns, however, do not have a direct relationship, and genes that vary in one measurement may not vary in the other since changes in mean expression do not translate to correlations. Given that previous works on gene expression and aging focused primarily on analyzing gene expression changes, it is relevant to ask whether genes that do not change expression levels with age may have altered co-expression patterns, as not necessarily a gene must change its expression to change a biological process.

Weighted co-expression networks, constructed with pairwise gene expression correlations, provide a robust framework for analyzing system-level changes between different conditions [13]. In these networks, genes are represented as nodes, and vertices indicate pairwise relationships between genes. These relationships – gene expression correlations – provide a weighted measurement of the strength of the interaction between two genes. Gene co-expression networks have been successfully used in analyzing biological data, including in the context [14–17] of aging.

Moreover, calculating specific network structural measurements can serve as metrics for comparing networks. One such metric is network connectivity, representing each gene’s sum of the network adjacencies (nodes). Therefore, connectivity in a gene co-expression network measures each gene’s general correlation with all other genes in the network. Another helpful metric is eigenvector centrality, which scores nodes based on the centrality scores of the other nodes they are connected to, such that a node with a high eigenvector centrality will be associated with nodes with high scores. Together, these network features reflect distinct biological information than gene expression levels.

Here, we analyzed changes in gene expression and co-expression networks with age using RNA-Seq data from eight tissues from the Genotype-Tissue Expression (GTEx) project [18]. Our results demonstrate that changes in gene co-expression reveal alterations that cannot be revealed by analyzing changes in gene expression alone, and that when both gene expression and gene co-expression data are taken together a more robust understanding of the regulation of gene expression during aging arises, showing conserved genes and biological processes, tied to specific protein complexes, being modulated with age across different tissues in humans.

## MATERIALS AND METHODS

### Dataset

Gene expression data (in TPM – transcripts per million) were obtained from the Genotype-Tissue Expression (GTEx) project. GTEx (v8 release) has postmortem RNA-seq data from 948 donors aged 20 to 70, collected from 54 tissues, totaling 17,382 samples. To increase the power of the analyses and allow the division of the dataset into age ranges, with an adequate number of samples in each age range, we selected only tissues with at least 800 samples (blood, brain, adipose tissue, muscle, blood vessel, heart, skin, and esophagus). Tissues were analyzed at the level defined by the SMTS sample attribute in the GTEx annotation files.

### Differential expression analysis with linear mixed model

Let Y_ijk_ be the expression level (log2 normalized TPM + 1) of gene *j* in sample *i* of individual *k*, *Age_i_* be the age of sample *i*, *Sex_i_* be the sex of sample i, *Race_i_* be the race of sample *i*, *Ischemia_i_* be the time of ischemia of sample *i*, *Batch_i_* be the experimental batch in which sample *i*’s gene expression was measured, *Death_i_* be the type of death, in the Hardy scale, of the donor from which sample *i* was collected, and *ε_ijk_* be the error term (assumed to be normally and independently distributed with var(ε) = σ^2^I). Then, we identified differentially expressed genes for each tissue separately using the following linear mixed model:


$$\begin{array}{l} Y_{ijk}=\beta_{0j}+\beta_{1j}Age_{i}+\beta_{2j}Sex_{i}+\beta_{3j}Race_{i}\\ \;\;\;\;\;\;\;\;+\beta_{4j}Ischemia_{i}+\beta_{5j}Batch_{i}\\ \;\;\;\;\;\;\;\;+\beta_{6j}Death_{i}+\beta_{7j}Individual_{k}\\ \;\;\;\;\;\;\;\;+\in_{ijk} \end{array}$$

Individuals are considered a block random effect, considering that some tissues have more than one sample for the same individual. Ischemic time was divided into 300-minute intervals to standardize the procedure with the removal of confounding factors, which was done in a later step. Random effects (Individual_k_) are assumed to be normally and independently distributed with var(*Individual*) = σ_ind_^2^I.

Genes with no or slight variations in expression were filtered out of the analysis. Selected genes had expression higher than 0.1 TPM in at least 20% of the samples. Depending on the analysis, genes needed to satisfy an FDR (false discovery rate) [19] <0.1 or <0.25 to be considered differentially expressed genes (DEGs) with age, considering BH multiple test corrections. The higher threshold of 0.25 was considered due to the rationale detailed in Results - Comparisons of altered genes across tissues. The significance of these higher threshold results is validated by the permutation tests described in Methods - Permutation tests for the intersection sets. The model was fit using the nlme package in R [20].

### Removal of confounding factors

For network analyses, expression was corrected for confounding factors using ComBat from the sva R package [21]. Since ComBat can only correct for one effect at a time, effects were removed iteratively, following the order of sex, race, experimental batch, ischemic time, and death type. Since ComBat does not accept continuous variables, ischemic time was divided into 300-minute intervals.

### Independence of connectivity and mean expression levels

The connectivity of a gene in a co-expression network is defined as the sum of that gene’s co-expression values with every other gene in the network. In co-expression networks, the co-expression between a pair of genes is defined as the correlation in their gene expression levels across samples (elevated to a soft-thresholding power in the case of WGCNA).

Let x_i_ and y_i_ be the expression levels of genes X and Y in sample i; $\bar{x}$ and $\bar{y}$ be the mean expression levels of genes X and Y across samples, and n be the total number of samples. Then,


$$cov(X,Y)=\frac{\sum_{i=1}^{n}\left(x_{i}-\bar{x})(y_{i}-\bar{y}\right)}{n-1}$$

is the covariance between X and Y, and


$$\sigma_{X}=\sqrt{\frac{\sum_{i=1}^{n}{\left(x_{i}-\bar{x}\right)}^{2}}{n-1}}\;\;\;\text{and}\;\;\;\sigma_{Y}=\sqrt{\frac{\sum_{i=1}^{n}{\left(y_{i}-\bar{y}\right)}^{2}}{n-1}}$$

are the standard deviations of X and Y. Finally, Pearson’s correlation coefficient (r) between a pair of gene expression vectors X and Y is defined as:


$$r_{X,Y}=\frac{cov(X,Y)}{\sigma_{X}\sigma_{Y}}$$

Consider a case in which gene Y is differentially expressed between two conditions, and a value of c increases its mean. If the increase of c is observed uniformly across all samples, the components in the formulas related to Y suffer the following transformation:


$$y_{i}-\bar{y}\overset{+c}{\to }(y_{i}+c)-(\bar{y}+c)=y_{i}-\bar{y}$$

Therefore, if the increase in expression is uniform across all samples, the covariances and standard deviations are unaffected. This way, the correlation between X and Y can remain the same in two conditions, even if Y is differentially expressed between the two conditions.

Similarly, correlations between X and Y can change, even if the mean expression values remain constant. Consider two extreme cases:

1. Y = X, with $\bar{y}=\bar{x}=\text{c}:$ all values of y in each sample i are identical to the values of x in the same samples, and means equal c;

2. Y = c, $\bar{y}=\text{c}:$ all Y values in each sample are identical and, consequently, equal to their mean c.

In case 1, the values of Y in each sample are variable and have a mean of c. Since Y and X are identical, their correlation is one. In case 2, Y values are constant and equal to c in each sample. Since Y remains constant as X changes, their correlation is zero. Less extreme cases can be easily imagined.

Therefore, gene expression correlations between pairs of genes can vary significantly between conditions, even if their mean expression values remain unchanged.

### Differential connectivity analysis

The dataset was divided into nine 6-year age ranges, except for the last one, which had a 3-year range. This division ensures that the smallest sub-dataset has at least 20 samples, the minimum recommended for WGCNA. Connectivity and eigenvector centrality for each gene were then calculated in each age range using the *igraph* R package [22]. For comparison between networks, the connectivities inside each age range were ranked, and a linear regression was applied to detect differences in ranked connectivities along the nine age ranges in the two metrics. Depending on the analysis, genes were considered differentially connected genes (DCGs) or differentially eigenconnected genes (DECGs) with FDR<0.1 or FDR<0.25. The higher threshold of 0.25 was considered due to the rationale detailed in Results - Comparisons of altered genes across tissues. The significance of these higher threshold results is validated by the permutation tests described in Methods - Permutation tests for the intersection sets.

### Permutation tests for the intersection sets

Intersection sets were tested for significance using permutation tests.

Consider G_ijk_ as the number of significantly altered genes in tissue *i* according to regression *j* (differential expression, differential connectivity, or differential eigenconnectivity) using an FDR threshold *k* (0.1 or 0.25). We constructed 100,000 permuted sets *p* (without replacement) of genes with the same size as each G_ijk_.

For example, for brain, we sampled without replacement 100,000 sets of 530 genes (number of DEGs at FDR<0.1), 100,000 sets of 7,699 genes (number of DCGs at FDR<0.1), and 100,000 sets of 9,421 genes (number of DECGs at FDR<0.1); with additional three sets for the cases with FDR<0.25.

The same process was repeated for all eight tissues, generating 100,000 sets of genes for each tissue, each of the three regressions, and each FDR threshold (8 tissues x 3 regressions x 2 FDR thresholds x 100,000 permutations). Therefore, each permutation *p* generated 48 sets, equivalent to all G_ijk_.

For each *p*, we calculated the intersections between tissues, considering all the scenarios analyzed. The proportion of permutations *p* with intersecting genes higher than the observed values was used to calculate a permutation p-value.

The higher threshold of 0.25 was considered due to the rationale detailed in Results - Comparisons of altered genes across tissues. However, despite this higher FDR threshold chosen for filtering tissue-specific altered genes, all intersection sets chosen for downstream analyses were significant at FDR < 0.001 in the permutation tests.

### Co-expression network construction and consensus module identification with weighted gene co-expression network analysis

Gene expression data for each analyzed tissue and age range without the effects of known confounding factors were used as inputs for the WGCNA package in R [23]. WGCNA constructs weighted gene co-expression networks using a soft threshold and fitting the data into a scale-free model. For each tissue, we selected the soft-thresholding power that resulted in networks in all age ranges within that tissue achieving an approximate scale-free topology index of 0.8. The chosen soft-thresholding powers for each tissue were: 6 (blood), 6 (brain), 9 (adipose tissue), 12 (muscle), 8 (blood vessel), 12 (heart), 6 (skin), and 6 (esophagus). We used the consensusBlockwiseModules function for module detection, with networkType set as “unsigned”. All parameters were kept at their default values. Sub-modules were defined by overlapping the positive and negative portions of the sets of genes of the three metrics (positive and negative DEGs, DCGs, and DECGs) with the modules defined by consensus modules analysis.

### Functional enrichment analysis

All enrichment analyses were carried out with the topGO R package [24], using the classic Fisher algorithm. Annotations for each gene were retrieved from Ensemble 110 [25]. Multiple test correction was performed using the BH method. Terms were considered enriched with FDR < 0.1.

For the enrichment of intersection sets, when the number of terms was too large to display correctly in the chart, we filtered by only showing the Biological Process terms and, if necessary, removing the terms with fewer genes annotated.

### Cross-tissue analysis of connectivity changes

To analyze how the same genes behave in different tissues regarding their connectivity, we only included the 14,489 protein-coding genes present in the analyses of the eight tissues (i.e., those that were not filtered out in any tissue during the initial data filtering stage). To facilitate cross-tissue comparison, we first reranked connectivities using only the protein-coding genes employed in this analysis (i.e., we removed all other genes and recalculated a new ranking in each tissue, including only the 14,489 genes analyzed here).

Our initial ranking included non-protein-coding genes in the calculation to account for their contribution to the information. Common, unfiltered non-protein-coding genes across tissues could have been included in this new ranking. However, given that we are only interested in protein-coding genes, we excluded them from the new rankings because non-protein-coding genes represent around half of the genes available in the dataset, and their connectivities are generally small compared to those of protein-coding genes, being heavily concentrated in the first half of the connectivity rankings. This feature caused significant distortions in the histograms, making visualization and interpretation of the behavior of protein-coding genes (our primary interest) difficult.

We created reference histograms for each tissue, which are histograms of the connectivity ranks of the DCGs for that tissue (referred to as the reference tissue). Given an x-axis representing all the connectivity ranks in bins of 100 ranks, the reference histograms show the connectivity distribution of all genes analyzed. Four reference histograms were constructed for each tissue: positive and negative DCGs in age range one and positive and negative DCGs in age range nine.

After constructing the reference histograms, other histograms were constructed. For each gene considered a DCG in a reference tissue, the connectivity ranks of these genes in different tissues (referred to as target tissues) are presented, regardless of whether they are also DCGs in these other tissues. In cases where DCGs are present in both the reference tissue and the target tissue, the regions representing them in the histograms are colored (red for positive DCGs and blue for negative DCGs).

Changes in correlations were tested for significance using the *cocor* R package [26], with the *cocor.indep.groups* function, using Fisher’s z.

### Comparison of enriched terms across sub-modules

To compare the results of the enrichment analyses between different sub-modules, we listed all enriched terms with FDR < 0.1 in any sub-module. We constructed a binary matrix with enriched terms in the rows and sub-modules in the columns. Entries had a value of 1 in the matrix if a term was enriched in the respective submodule. The terms were then filtered. First, terms found in fewer than four tissues were removed to focus on processes common across tissues, resulting in a final list of 2,310 terms. Secondly, terms found in the first four and last six levels of the ontology tree from the Biological Process Gene Ontology tree were removed, with the rationale that this strategy removed terms that were too general or too specific to yield relevant biological information. Finally, we removed all the columns (sub-modules) that displayed no entries after the previous filtering. After filtering, 1,956 terms were included in the matrix. These steps ensure that: i) our matrix of terms per sub-module is less sparse (with a higher density of 1 values), making it easier to cluster, interpret, and present (due to its smaller size); and ii) reduce the number of terms to a smaller value that is within the limit of the tool we used for the analysis of semantic similarity (Revigo [27]), which accepts at most 2,000 terms as input.

This binary matrix was then clustered into 8 clusters using the *hclust* function from the stats package in R [28], using the *Ward.D* clustering algorithm [29]. The results were presented as a heatmap using the *heatmap.2* function from the *gplots* package in R. The same list of enriched terms that comprise the heatmap rows was then summarized using the online tool Revigo [27]. Revigo summarizes lists of Gene Ontology terms based on their semantic similarities and creates a chart where the distances between points represent their semantic distances. Next to the terms, we included the number of tissues each term is enriched in (considering any sub-module in that tissue). Our list of terms was summarized using the following parameters in Revigo: *SimRel* semantic similarity algorithm, “Small” option for the size of the final list, “Yes” for removing obsolete terms, “*Homo sapiens*” for the species, and “Higher value is better” for the interpretation of the additional value (in our case, the number of tissues in which the enriched term is present). The R code for reproducing the chart was then downloaded, and, within R, a variable containing the ID of the cluster in which the hclust function clustered that term was added. The final chart was plotted using the number of tissues as a variable to define the size of the points and the cluster variable to define the coloring of the points.

### Protein-protein interaction (PPI) network analysis with STRING

The PPI networks were analyzed using STRING [30], which utilizes the STRING extension for Cytoscape [31]. For constructing our networks, we used scores of at least 0.7, which are considered high in STRING. We queried genes in the 427 set and the 134 set not already included in the 427 set (these sets represent the sets of genes that change in common across 7 and 8 tissues, and how they were selected is described in *Results – Comparison of altered genes across tissues*), for a total of 506 genes, of which 505 were identified in the STRING database.

### Gene overlaps

Gene overlaps and overlap significance statistics were calculated using the *GeneOverlap* R package [32].

### Data availability

The raw data used in this study’s analyses are available through the Genotype-Tissue Expression (GTEx) project at gtexportal.org/home.

## RESULTS

### Detection of tissue-specific differentially expressed and connected genes

For this study, we used data from the GTEx project (version 8), which has whole transcriptome data for up to 54 tissues from 948 postmortem donors. To maximize statistical power, we aggregated sub-tissues and selected only tissues with at least 800 samples each. We then analyzed the blood, brain, adipose tissue, muscle, blood vessels, heart, skin, and esophagus data. The data was processed as described in Methods. Figure 1A shows a general schematic of data processing and analysis.

**Figure 1:**
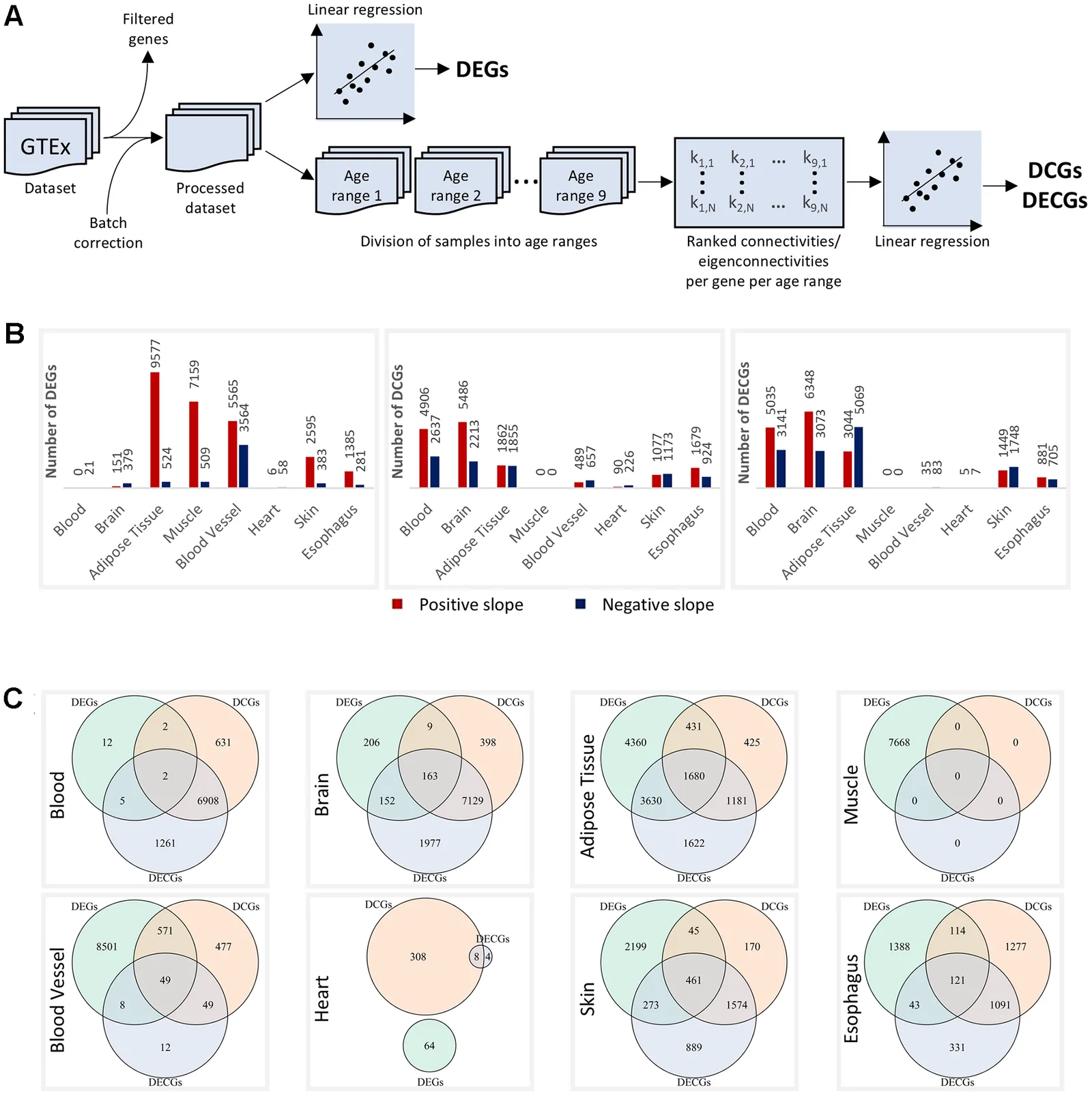
**Altered genes per tissue.** (**A**) Schematics of the differential expression and differential connectivity/eigenconnectivity analyses. (**B**) DEGs, DCGs, and DECGs per tissue. The red and blue bars represent genes with positive and negative slopes, respectively. All metrics considered FDR < 0.1. (**C**) Overlaps between DEGs, DCGs, and DECGs in each tissue.

The differential expression and connectivity analyses were then carried out as described in the Methods section. For the differential connectivity analyses, the dataset was divided into nine age ranges (Supplementary Figure 1), and networks were constructed using WGCNA [23] (Methods). All results obtained from the three regressions are presented in Supplementary Data 1. For all metrics, genes were considered altered with FDR < 0.1.

The number of differentially expressed genes (DEGs), differentially connected genes (DCGs), and differentially eigenconnected genes (DECGs) varied considerably by tissue (Figure 1B). Notably, many tissues demonstrated relative stability in either the differential expression analysis or one of the connectivity analyses. Blood, brain, muscle, and blood vessels all clearly fall into this category, having many DEGs or DCGs/DECGs, but not both simultaneously. Adipose tissue had many significant genes in all analyses, but a clear tendency towards having more DEGs than DCGs can be seen. Only the heart, skin, and esophagus had relatively similar numbers of DEGs and DCGs/DECGs (though very few in the heart).

Only a small proportion of genes were simultaneously DEGs and DCGs/DECGs (Figure 1C). The p-value distribution of the expression and connectivity regressions against one another shows a significant discordance between the metrics (Supplementary Figure 2A). A similar behavior is seen when comparing the expression regression with the eigenconnectivity regression (Supplementary Figure 2B). On the other hand, the connectivity and eigenconnectivity regressions show remarkable concordance (Supplementary Figure 2B), suggesting these metrics measure similar biological effects.

These results demonstrate that even in tissues with a high number of significant genes across all metrics—such as adipose tissue, skin, and the esophagus—there is a clear tendency for the genes that alter gene expression levels to remain unchanged in one of the connectivity metrics, and vice versa.

### Comparisons of altered genes across tissues

To compare the genes found in each tissue, we counted the number of tissues in which each gene was changed in each metric. We define sets of genes changing in common across different tissues as intersection sets. The results for DEGs and DCGs are shown in Figure 2A.

**Figure 2:**
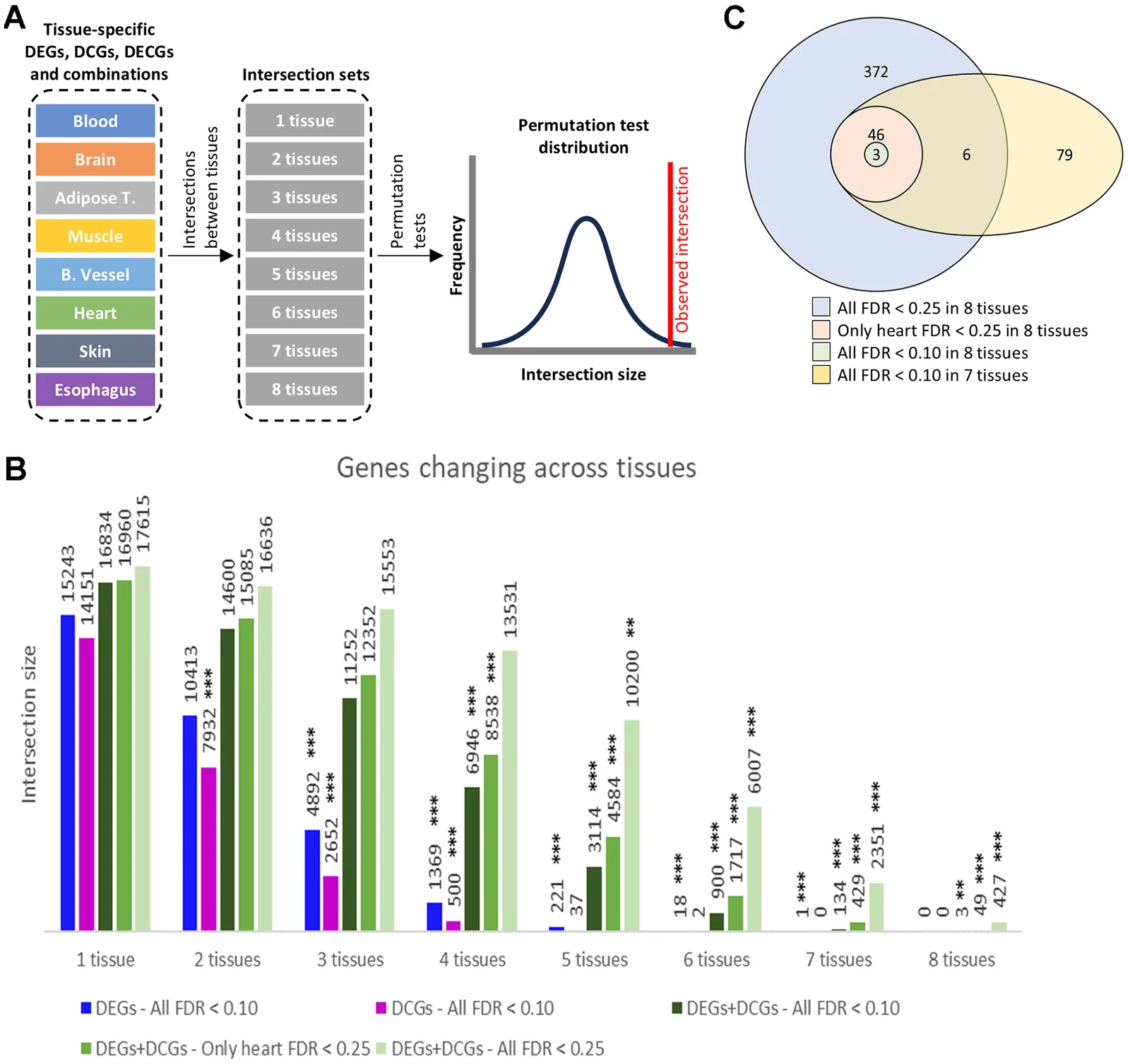
**Intersect count.** (**A**) DEGs, DCGs, and their combination, detected in sets of 1 up to 8 tissues. (**B**) DEGs, DCGs, and their combination, detected in sets of 1 up to 8 tissues. The charts display the number of genes in the set and their significance in a permutation test. Significance levels: FDR<0.1 (*), FDR<0.01 (**), FDR<0.001 (***). (**C**) Venn diagram of selected intersection sets.

Most intersection sets are significant considering permutation tests (see Methods). Non-significant sets are either in very few tissues (DEGs in 1 and 2 tissues and DCGs in 1 tissue) or in tiny sets in many tissues (DCGs in 5 and 6 tissues). Results, including DECGs, are similar (Supplementary Figure 3).

The non-significance for the sets in one tissue is expected, as they are not true intersections and only indicate that most genes are altered in at least one tissue, demonstrating the heterogeneous component of tissue aging.

Given the discordant nature of the differential expression and differential connectivity regressions (Supplementary Figure 2A), we asked whether genes that are DEGs in some tissues might be DCGs or DECGs in other tissues. This is relevant because both analyses detect changes in transcriptomic behavior, even if the changes are different and independent (see Methods section for a demonstration of the independence between the two metrics). We then considered four scenarios:

1. Whether the gene changed in any metric (DEG/DCG/DECG);

2. Whether the gene is a DEG and/or a DCG;

3. Whether the gene is a DCG and/or a DECG or

4. Whether the gene is a DEG and/or a DECG.

The results show that although most genes changing with age are tissue-specific or shared between a few tissues, when intersections between tissues are considered using different metrics to assess transcriptomic behavior, a core of common genes changes across tissues (Figures 2A and Supplementary Figure 3).

Interestingly, gene sets in scenarios including DEGs and one of the connectivity metrics (DCGs or DECGs) in many tissues were significant, including in eight tissues. Sets that include DCGs and DECGs simultaneously, however, were insignificant. This can be understood considering that DCGs and DECGs overlapped significantly (Supplementary Figure 2B), which makes considering both partially redundant, while permuted sets would be more significant (see Methods).

The small intersection set in eight tissues is caused by the heart. Interestingly, the distribution of raw p-values in the heart does not match what would be expected if the null hypothesis were true for all genes (i.e., none are changing) (Supplementary Figure 4), as can be observed for blood in the differential expression regression (Supplementary Figure 4).

While a typical scenario of FDR correction with so many tests when there are close to no significant genes can be observed in the FDR density histogram for blood in the differential expression regression, the same density histogram in the heart displays a very different pattern, with most FDRs clustering around 0.20-0.25. Additionally, when we plot the FDR distribution of genes belonging to the three sets encompassing more tissues using FDR < 0.1, they cluster around the 0.20-0.25 range in the heart (Supplementary Figure 5). This pattern strongly suggests a non-random distribution of p-values and raises the question of whether the genes that cluster around 0.20-0.25 FDR in the heart are meaningful.

To analyze this, we considered two additional scenarios for the intersection analysis, where we raised the FDR threshold to 0.25:

(i)Considering genes significant at FDR < 0.25 only for the heart and

(ii)Considering FDR < 0.25 for all tissues.

It is essential to note that while increasing the FDR threshold increases the probability of including tissue-specific false positives, this shortcoming is mitigated in the next step of the analysis, where we assess the significance of the intersection sets. Essentially, an actual false positive would be a completely random gene that was below the FDR threshold due to chance alone. However, if the same gene was below the FDR threshold (0.10 or 0.25) in 7 or 8 tissues, then it is extremely unlikely that we are observing a false positive. In other words, true randomness would cause random genes to cross the threshold in different tissues, resulting in very low cross-tissue intersections. So, if a sufficiently large number of genes are crossing the threshold in 7 or 8 tissues, then they are extremely unlikely to be random false positives.

To put it in a slightly different way, any gene that was a false positive in one tissue due to a higher FDR threshold would likely not end up in the final intersection sets, especially in those intersection sets in 7 or 8 tissues, since it would have to coincidentally also be a false positive in all of these other tissues. In essence, the intersection sets in a high number of tissues ultimately exclude false positives. This means that our goal here should be to test whether the number of genes in the intersection sets is large enough to rule out the probability of them being coincidentally false positives.

Indeed, both additional scenarios yielded significant sets in 5, 6, 7, and 8 tissues (Figure 2A); of particular interest are the sets in 8 tissues for both scenarios, with 49 and 427 genes, which represent conserved sets of genes that display transcriptomic alterations across all tested tissues during aging. Both were significant at FDR < 0.001 in the permutation tests, indicating that despite using a higher FDR threshold (0.25) to filter tissue-specific results, the resulting cross-tissue sets are highly significant.

Since the results for the connectivity and eigenconnectivity analyses were well correlated, we focus on DEGs and DCGs in most analyses. Henceforth, we will refer to the sets of genes presented in Figure 2A as “intersection sets,” the specific sets of interest (with the combination of DEGs and DCGs) will be called 3-set, 49-set, 427-set, and 134-set. Figure 2B shows a Venn diagram overlapping these sets of interest.

### GO enrichment analysis of intersection sets

We performed a GO enrichment analysis of the intersection sets using the *topGO* R package. Given that we are not interested in tissue-specific changes but rather in intersection sets across most tissues, we limit the results to intersection sets comprising fewer than 5,000 genes, as shown in Figure 2A. Supplementary Data 2–4 contain enrichment results for all these sets.

In Figure 3, we show the results for selected intersection sets. The sets shown are those with the most tissues for which enrichment results identified significant terms in the Biological Process ontology, accompanied by a reasonable number of essential genes annotated.

**Figure 3:**
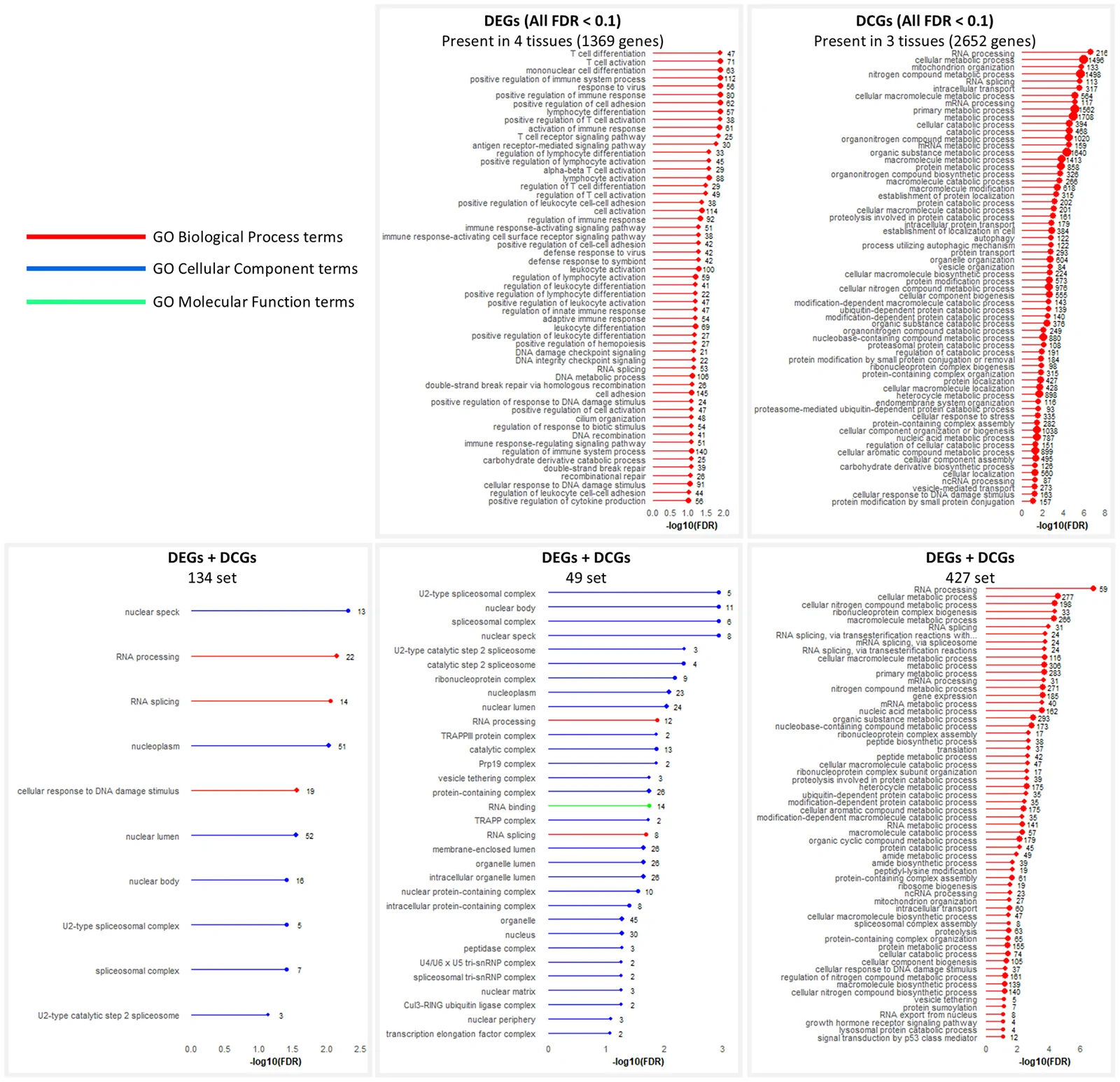
**GO enrichment analysis of intersection sets.** Terms significant at FDR < 0.1. In cases where too many terms were enriched, only Biological Process terms are shown. When that still yielded too many results, further filtering was performed by removing terms with the least number of genes.

While the DEGs chart displays immune system-related terms not found in the DCGs chart, they both contain terms related to RNA splicing and DNA damage response. Furthermore, while the set of DCGs in 4 tissues was not chosen because of the previously mentioned criteria, its analysis shows enrichment in Cellular Component terms for the spliceosomal complex (Supplementary Data 2). This indicates that four tissues display good evidence for enriching intersecting DEGs and DCGs related to RNA splicing and DNA damage response.

The selected intersection sets in seven or eight tissues show similar results, with enrichment in RNA splicing, RNA processing, and response to DNA damage. The FDR distribution of all RNA splicing and processing genes corroborates the tendency of these genes to be located in the areas of greatest significance in the charts of all tissues. It reinforces the tissue-specific discordant behavior of the genes in the two regressions (Supplementary Figure 6).

It is important to note that the term “RNA splicing” is a direct child of the term “RNA processing” in the Biological Process ontology tree, meaning that RNA splicing genes are contained in the RNA processing set.

DNA damage response is not found in the 49-set but is in the 427-set, along with terms related to protein catabolism, autophagy, and ribosome biogenesis. Supplementary Figure 7 displays the induced GO subgraph of the top 20 enriched terms in the 134-set.

In most tissues, genes in these sets are predominantly upregulated in at least one of these two metrics (Supplementary Figure 8), except for blood vessels, which have a mixed profile, and the heart, in which most genes are negative DEGs.

Furthermore, negative DEGs, including those in the 427-set, tended to be highly expressed in younger samples (Supplementary Figures 9, 10). In fact, in blood vessels and the heart, where there was a higher proportion of negative DEGs, both overall and in the intersection sets, the negative DEGs remained highly expressed in older samples, even with the decreases, indicating that most negative DEGs remain necessary for cellular function.

Changes in connectivity between young and old samples tended to be more dramatic, with DCGs in the 427-set and the splicing genes tending to become more highly connected at older ages (Supplementary Figures 9, 10). The most apparent exception seems to be skin, where most splicing and 427-set genes decrease their connectivities (Supplementary Figures 8, 9). The 134-set and the 49-set show similar results (Supplementary Figure 10).

### Cross-tissue analysis of connectivity changes

It is puzzling that some tissues tend to have more DEGs or DCGs but not both. Even in the skin and esophagus, where the number of DEGs and DCGs was more balanced, the genes changing in one metric did not change in the other. To explore this phenomenon, we analyzed the distribution of DCG connectivity ranks in each tissue against the connectivity ranks of the same genes in the other tissues (see Methods).

The results indicate that positive DCGs in each tissue tend to be among the lowest or middle-ranked genes in their tissues in younger samples (Figure 4A). However, when we examine the connectivity rank histogram of these same genes in other tissues, we observe a contrasting scenario, with the same set of genes being significantly more highly ranked in the different tissues. For example, positive DCGs in the blood are among the low and middle ranks regarding connectivity. However, the same genes that are positive DCGs in blood tend to be more highly ranked in other tissues.

**Figure 4:**
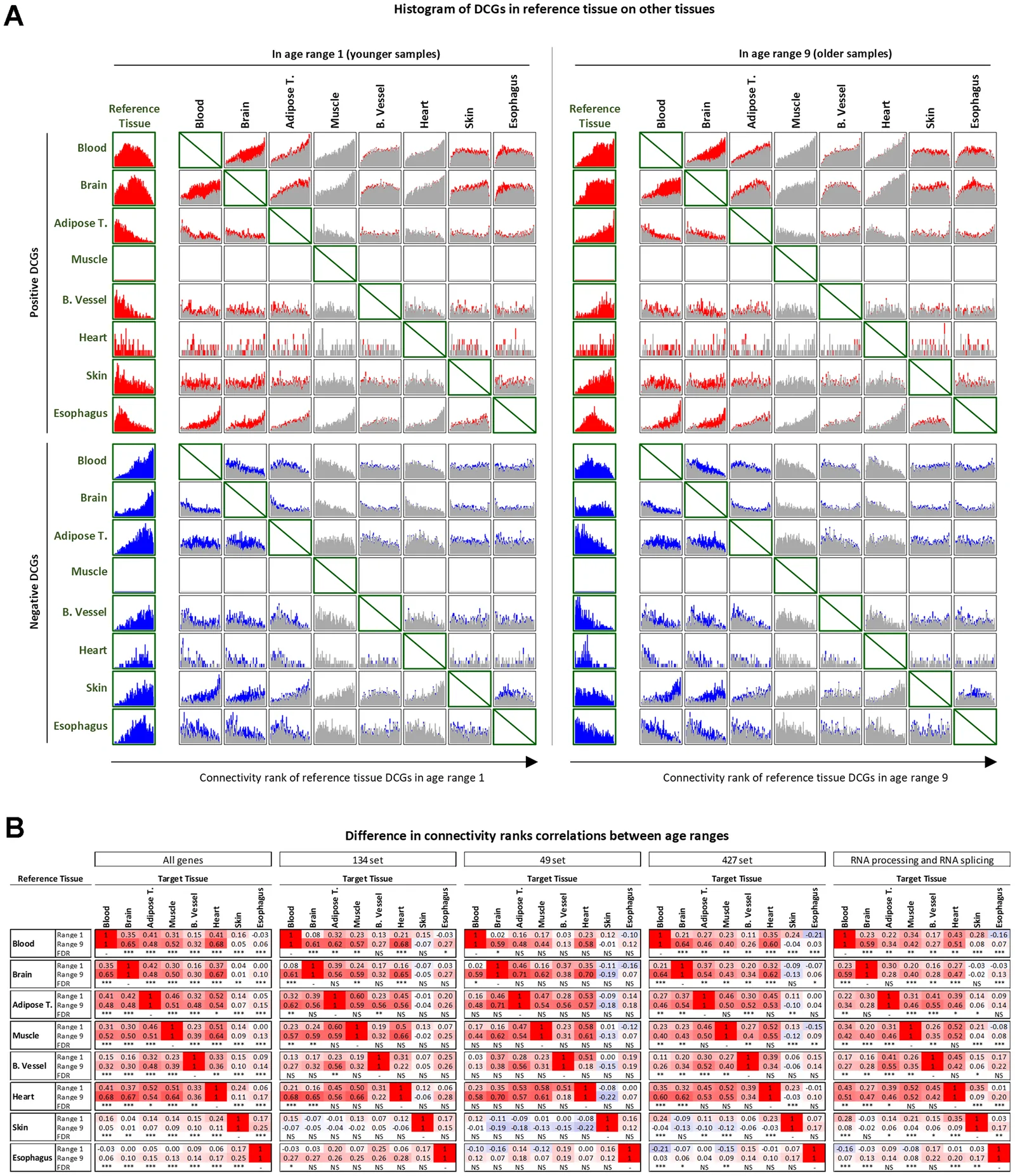
**Cross-tissue analysis of DCGs.** (**A**) DCGs in reference tissues projected on other tissues in younger and older samples. Colored portions of the histogram represent positive (red) or negative (blue) genes DCGs in the reference and target tissue. Grey portions represent genes that are DCGs in the reference tissues but not in the target tissue. (**B**) Comparison of changes in correlations of connectivity ranks between younger and older samples. Significance levels: FDR<0.1 (*), FDR<0.01 (**), FDR<0.001 (***).

Negative DCGs show similar behavior with inverse logic. The only exception is skin, since its negative DCGs are highly ranked in many other tissues.

In older samples, the DCGs changed their rankings as expected, such that the connectivity ranking pattern of these genes more closely resembles the pattern they already displayed in other tissues. This suggests that gene connectivity is reorganized in some tissues, and that in tissues where these genes are not changing their connectivities, it is likely because their connectivities were already roughly at the physiologically ideal level.

This convergence of connectivity ranks is evident when comparing the correlations of connectivity ranks between pairs of tissues in the younger and older age ranges. Correlations are consistently significantly increased in all tissues, except correlations involving the skin, which are consistently considerably decreased (Figure 4B – first panel).

A similar pattern can be seen when considering only the intersection sets or all RNA processing and RNA splicing genes (Figure 4B—second to fifth panels). In these cases, however, the reduced number of genes makes it more challenging to determine the significance of all comparisons, especially for the 49-set, even when the increase in correlation is significant (for example, see the comparison between brain and muscle in the 49-set).

### Consensus network and GO enrichment analysis of age-related sub-modules

While only a few common genes appear to be changing across 7 or 8 tissues, we inquired whether other genes changing in fewer tissues could exhibit similarities at the process level.

We used WGCNA to build an unsigned consensus network for each tissue’s nine age ranges. This allowed us to identify conserved gene co-expression modules that were present in all nine datasets. After obtaining these modules, we overlapped them with DEGs, DCGs, and DECGs and divided them into positive and negative portions. This led to the definition of sub-modules, where each sub-module comprises the positive or negative DEGs, DCGs, or DECGs contained in that module.

By dividing the DEGs, DCGs, and DECGs using the modular structure provided by WCGNA, we group them into sets likely to be related to the same biological processes and, therefore, identify them based on a functional enrichment analysis.

Since enriching these sub-modules generated many lists of terms, we first summarized them in a heatmap format (Figure 5A and Supplementary Figure 11). Then, we used Revigo to display the results (Figure 5B) (see Methods for a detailed description). Complete enrichment results per module can be found in Supplementary Data 5–7.

**Figure 5:**
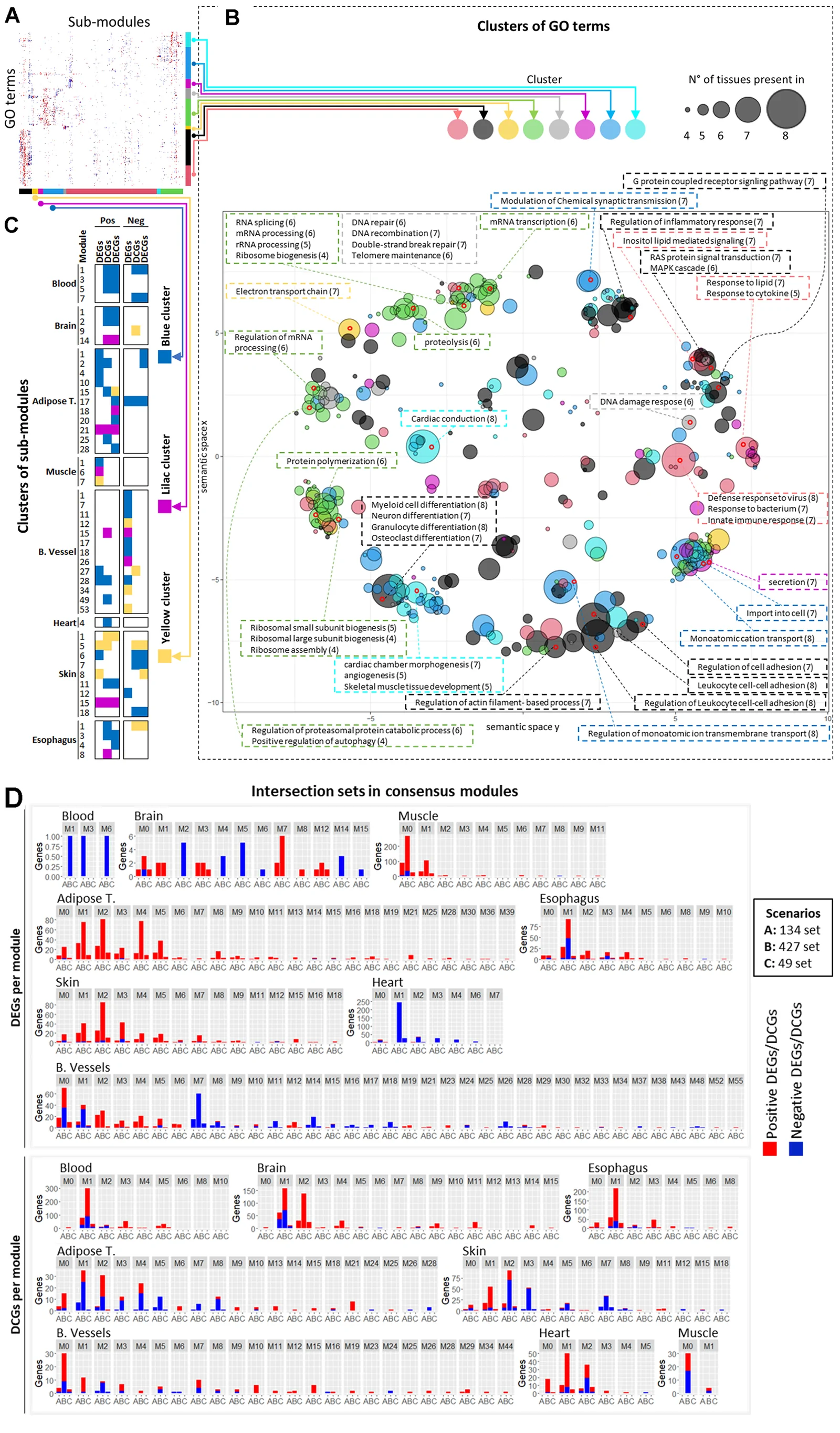
**Consensus modules analysis.** (**A**) Unlabeled heatmap of similarity between terms enriched in sub-modules. (**B**) Semantic similarity of the terms in the heatmap with Revigo. The size of the dots on the chart represents the number of tissues in which that term is found, and the coloring of the dot represents the cluster of terms/rows in which that term is found. The size and coloring are based on the term chosen to represent the cluster. Terms contained in some points in different regions of the chart are highlighted and described in the text boxes. The numbers in parentheses next to the terms represent the number of tissues in which that term is enriched (which may or may not be equal to the value for the representative term of the same point). (**C**) Sub-modules enriched in terms contained in the green and gray terms/rows clusters. (**D**) Overlap between consensus modules and genes in the intersection sets. The red and blue portions of the bars represent positive and negative DEGs/DCGs.

The green rows/terms cluster contains sub-modules enriched in RNA splicing and processing, the same processes enriched in the intersection sets (Figure 5A and Supplementary Figure 11). Inspecting the heatmap and Revigo results (as well as Supplementary Figure 11 and Supplementary Data 5–7) show that, in addition to the RNA splicing and processing terms, other processes were frequently enriched in the sub-modules in the same green cluster, such as proteolysis, protein catabolism, autophagy, and ribosomal biogenesis.

Additionally, the grey cluster displayed a considerable overlap in sub-modules with the green cluster and contained terms related to DNA repair and DNA damage response. This suggests that these processes change in a coordinated fashion during aging, which was already indicated with the enrichment of the 427-set (Figure 3).

These terms’ sub-modules are grouped in the blue, lilac, and yellow columns/submodule clusters (Figure 5C).

Subsequently, we counted the number of DEGs and DCGs in the intersection sets in each module in each tissue (Figure 5D). Most genes in the intersection sets are grouped in the first (and largest) modules, which contain sub-modules in Figure 5C.

Only the heart and skin display different behaviors. This is expected in the case of heart since the sub-modules were defined using only DEGs and DCGs with FDR < 0.1, which were very few.

Only M7 submodules related to skin are found in the blue cluster (Figure 5C). Almost all M2 submodules, which concentrate more genes in the intersection sets, had no enriched terms (Supplementary Data 5–7), explaining why they were not clustered in Figure 5A.

M1 and M5 skin submodules are present in Figure 5C but are contained in the yellow submodule cluster, which, although it has a pattern of terms similar to the blue submodule cluster, has a higher number of terms from the gray cluster, related to DNA repair and response to DNA damage.

The M3 skin module has enriched terms in the submodules for positive DEGs, negative DCGs, and negative DECGs (Supplementary Data 5–7). However, they are more enriched in terms of ribosomal biogenesis, translation regulation, and RNA processing. Only in the submodule of negative DECGs do terms related to RNA splicing appear enriched at FDR < 0.1.

These results show that although the genes in the intersection sets were the only ones changing across tissues, they associate with modules that contain other genes that are changing in a tissue-specific manner, related to similar processes, which act in a coordinated fashion with the genes in the intersection sets.

### Protein-protein interaction prediction network analysis

Co-expression relationships do not identify the molecular mechanisms through which biological interactions manifest. Therefore, to determine whether there are physical associations between the protein products of these genes, we constructed protein-protein interaction (PPI) networks using STRING. The STRING database has curated and predicted protein-protein interactions based on several inferences.

Several proteins in the analysis have strong evidence of being part of physical complexes (Figure 6A, 6B). Many of them cluster into very well-defined physical complexes. As expected from our previous results, several interact in the spliceosome.

**Figure 6:**
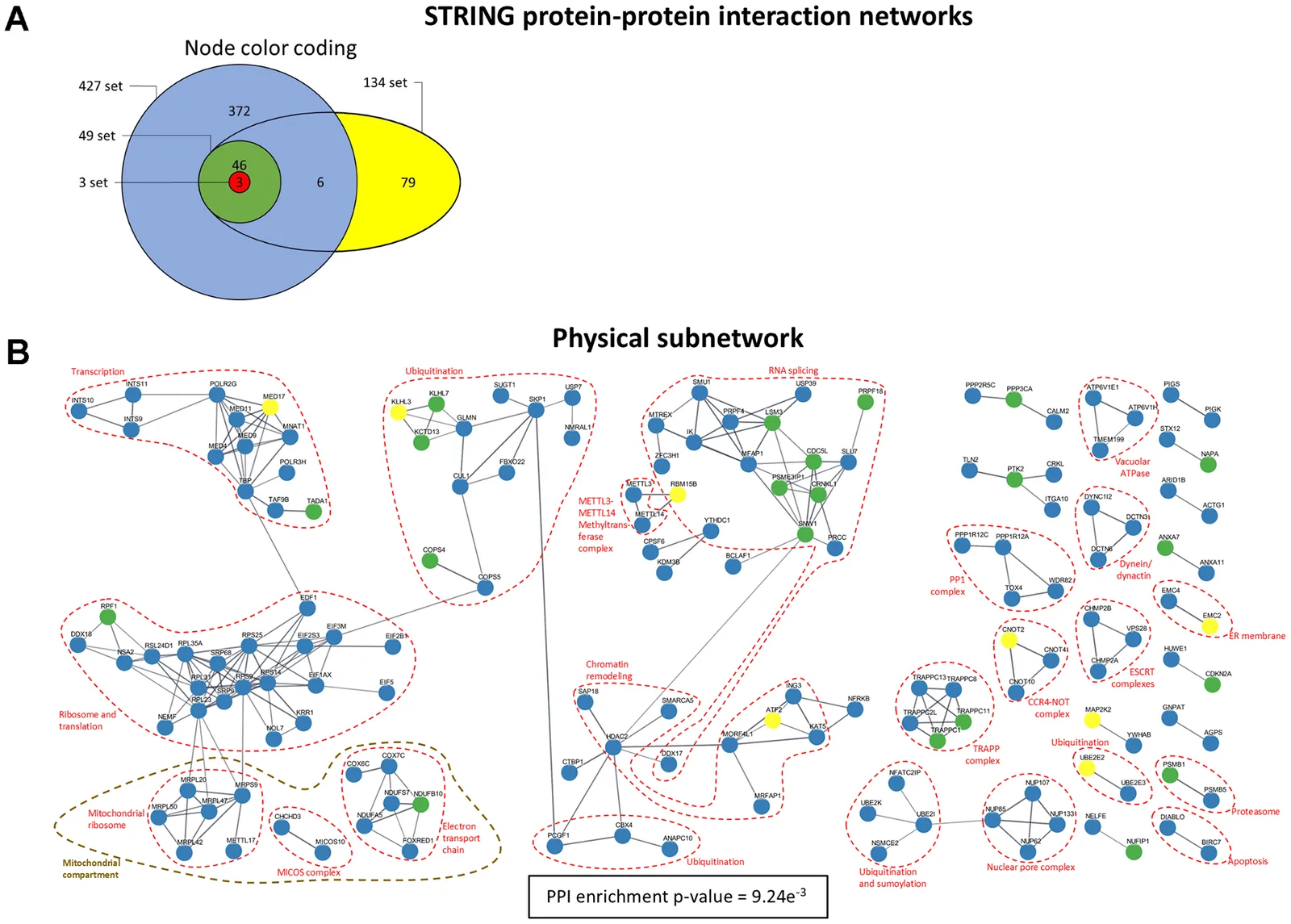
**STRING PPI physical subnetwork.** (**A**) Color coding for the nodes in the network indicating to which intersection set the corresponding genes belong. (**B**) Physical subnetwork. The network includes links with a confidence score of at least 0.7 in STRING. Singletons (unconnected nodes) are omitted.

Additionally, many ribosomal proteins (both cytosolic and mitochondrial) are present, along with translation initiation and elongation factors. Several proteins involved in transcription and chromatin remodeling, as well as proteins involved in ubiquitination and proteolysis, are clustered. The PP1 complex highlighted is also involved in regulating RNA splicing [33].

The complexes identified in this analysis delineate a continuous path, with the transcription of mRNAs being followed by RNA splicing and processing and subsequent translation in the ribosomes, after which proteins are sent to the ER. During this process, damaged or misfolded mRNAs and proteins are degraded. The degradation of proteins is represented in the network by proteins that promote the ubiquitination of other proteins and tag them for degradation, as well as some protease-related proteins. In mitochondria, in addition to the mitochondrial ribosome, some proteins are components of the electron transport chain. Interestingly, only components of Complexes I (NADH dehydrogenase) and III (cytochrome c oxidase) seem to be altered. Similar results are seen in the whole network (Supplementary Figure 12).

## DISCUSSION

Several studies have attempted to identify similarities in gene expression across tissues using different strategies [6–12, 34, 35], including employing WGCNA [14, 17]. Still, no common patterns were found at the gene level, and very few were found at the process level.

Our work’s initial premise was that differential expression analysis alone cannot detect all alterations that occur in the transcriptome with age, and that a lot of information is lost when only changes in absolute expression levels are considered. This lost information may be able to explain why there has been so much difficulty in finding clear common patterns in transcriptomic changes with aging across tissues, especially in terms of individual genes. We hypothesized that part of this information may be contained in changes in co-expression (reflected in network topological metrics), under the assumption that some genes may not display differences in absolute gene expression levels with age but may change the patterns with which they co-express with others. This combined approach yielded very different results from a traditional differential expression analysis, revealing patterns of transcriptomic changes that cannot be seen by looking only at absolute gene expression values, as even tissues with very few DEGs displayed a considerable amount of DCGs and DECGs, and vice versa.

Our results demonstrate that a core of genes is being altered across tissues, and this core is significantly enriched in RNA splicing and processing terms, in addition to being well-connected in PPI networks. However, these changes are captured by differential expression or differential connectivity analyses, but rarely in both.

This demonstrates that looking only at differential expression may lead to an incomplete picture of the age-related transcriptomic adjustments some tissues undergo. For instance, given the preponderance of DCGs in blood and brain, it is no surprise that no common set of DEGs would be found between them and other tissues. Any analysis comparing only DEGs using these two tissues would miss the reality that these tissues display transcriptomic changes with age reflected much more heavily in co-expression/connectivity-related changes than in absolute expression changes. Therefore, ignoring the differences in connectivities in some tissues may lead to a false notion that they do not display age-related transcriptomic changes in a common set of genes.

Additionally, we believe that the results obtained by relaxing the FDR threshold to 0.25 in the first step of the analysis, and then verifying the significance of the intersection sets in the subsequent step with permutation tests using stricter thresholds, reinforces our results, since a pattern emerges regarding who is included in the intersection sets as the FDR threshold is increased.

Making an analogy, one way to think of this is to imagine the entire biological network and picture an imaginary circle within it that contains a small cluster of well-connected genes that change in common across tissues according to a certain significance threshold, and which seems to be primarily enriched in RNA splicing. As the significance threshold is increased, this circle expands, encompassing genes that were neighbouring the initial small cluster, which includes (i) more RNA splicing and processing genes, (ii) more genes in processes immediately upstream and downstream to RNA splicing, and (iii) more genes whose protein products are well connected to the previous PPI network. This denotes a non-random pattern of inclusion, which has a clear biological meaning.

The contrasting scenario would be if increasing the significance threshold led to other random circles popping up all around the biological network, indicating no clear pattern. This would be expected if the inclusion of genes in the analysis, due to the higher FDR threshold of 0.25, had resulted in false positives. It would require an extremely improbable data distribution to generate this clear pattern of inclusion if the significant genes at FDR 0.25 were purely the result of chance. Therefore, this pattern of inclusion when increasing the FDR threshold reinforces the results. When this logic is considered, the question ceases to be whether there is a common set of genes related to specific enriched processes changing across tissues with aging, and a new question arises as to why these different tissues undergo transcriptomic changes in varying ways.

At least for DCGs, differences can be explained mainly by the fact that the connectivities of genes in each tissue are reorganizing so that their rankings converge with age, with skin being the only exception. This behavior could indicate that these changes reflect responses to a common stimulus. Supposing the burden of defective spliced RNAs and malformed proteins increases with age, it seems reasonable for all tissues to reorganize themselves to deal with this problem. However, given that different tissues will have different starting points regarding the connectivity of each gene, it makes sense that how many genes will change in a given tissue and the trajectory of these changes will be different according to tissue, even if the endpoint might be approximately the same. Some tissues might already have a specific configuration of connectivities of their genes at young ages that allow them to deal with higher burdens of aberrant splicing and malformed proteins. In contrast, others may be more susceptible to this alteration and may need to promote a more robust reorganization of their molecular network to deal with the increased burden of age-related protein malformation. Therefore, a standard stimulus may condition different tissues to converge to a similar adaptive state.

One possibility for the different behavior observed in the skin might be that it is the only tissue analyzed that is directly exposed to the external environment, with a greater impact of exogenous factors than in other tissues, creating a different pattern of reorganization. Nonetheless, there is mostly upregulation of DEGs in the intersection sets for skin, indicating that the burden of defective spliced RNAs may indeed be greater. The fact that many negative DCGs in the skin are also positive DEGs (Supplementary Figure 8), including RNA processing and splicing genes, indicates that they require an increased activity of these processes with age. Furthermore, a significant subset of genes in the intersection sets and RNA splicing sets are positive DCGs, indicating, to some extent, a greater necessity for these genes.

Alternative splicing is a fundamental process in eukaryotes that allows the same gene to encode multiple different transcripts, with approximately 95% of multi-exon genes producing transcripts that undergo alternative splicing [36]. Therefore, it is intuitive that alterations in the splicing machinery would have systemic effects on the biological network. Additionally, alternative splicing is a crucial mechanism for determining tissue identity and homeostasis, enabling the same genes to express different isoforms in various tissues according to the specific tissue’s needs [37, 38]. This tissue-specific character of splicing means that common cross-tissue alterations in the splicing machinery can lead to different downstream effects in different tissues, possibly explaining tissue-specific signatures of age-related gene expression.

Indeed, aging appears to be accompanied by a high incidence of aberrant splicing and intron retentions in *C. elegans*, fruit flies, mice, and humans [39–44]. Additionally, differential expression of splicing factors has also been found to be associated with aging [39, 44–49]. Changes in alternative splicing are also observed in several age-related diseases [49]. Modulation of specific splicing factors has been shown to increase lifespan in model organisms [50, 51], and splicing appears to be modulated in model organisms during dietary restriction and mTOR inhibition [41, 42], two known lifespan-promoting interventions. Spliceosome activity also seems to have a role in the regulation of cellular senescence [52], a process that is associated with aging. Notably, Mariotti et al. (2022) [43] showed an increase in these events in the same GTEx dataset for several tissues. Most tissues they analyzed showed an increase in intron retention with age. This includes heart and blood vessels, for which we detected decreased RNA processing/splicing expression in the intersection sets. One possible explanation for the observation that these tissues have a higher aberrant splicing burden, but less expression of splicing genes, is that the changes in connectivity ranks are taking the lead in adapting these tissues to this alteration. Since both tissues display a convergent pattern of connectivity ranks, these changes may already be boosting the adaptation to aberrant splicing as much as the tissue can. Furthermore, the negative DEGs in the intersection sets do not result in a dramatic decrease in expression.

Determining the cause of this increased incidence of aberrant splicing is currently impossible. One hint is the presence of genes associated with DNA repair and DNA damage response. The idea that DNA alterations cause aging is one of the most classic theories of aging [53, 54], and DNA damage is a hallmark of aging [55]. It is possible, therefore, that damage to DNA is leading to aberrant splicing. Indeed, there seems to be a connection between RNA splicing and DNA damage response [56], even at the transcriptional level. Furthermore, previous studies have shown that the expression of DNA repair genes is positively associated with species’ maximum lifespans [57–59], and that transcriptional signatures related to maximum lifespan seem to present similarities with those seen during aging in a tissue-specific manner [60].

The processes clustering with RNA processing/splicing in the same modules are interesting because they all coordinate during the protein production cycle. They can be easily interpreted in light of what we know about aging and the lifespan extension promoted by mTORC1 inhibition. Inhibition of mTORC1, through genetic or pharmacological means, is one of the most studied methods of life span extension and works in several organisms, including mice [61–76]. mTORC1 is a central regulator of autophagy [77]. Therefore, higher expression and relative connectivity of autophagy and proteolysis/protein catabolism genes, as indicated in our enrichment results, demonstrate that these mTORC1-related processes are more prominent during aging (see Figure 5 and Supplementary Data 5–7). Loss of proteostasis is also a hallmark of aging [55], and aberrant splicing is a cause of malformed proteins, which demand more protein degradation mechanisms to remove these malformed proteins.

Lastly, ribosomal biogenesis has also been associated with aging [78]. The presence of these processes in the modules found here also suggests that they work in a coordinated fashion. Higher rates of splicing abnormalities may lead to more malformed proteins, which will demand more protein degradation machinery. Given the folding challenges, such aberrant mRNAs may create difficulties for the ribosomal machinery since they may take longer to translate. Therefore, we could speculate that this increased occupancy of ribosomes would end up signaling for the biogenesis of more ribosomes to decrease the amount of mRNA in line to be translated.

From the results described here, it is reasonable to imagine a scenario in which the age-associated increase in aberrant mRNAs, proteins, and eventually organelles, which were negatively affected by malfunctioning proteins, may impose significantly on catabolic processes such as RNA catabolism, protein catabolism, and autophagy. Lifespan extension by mTOR inhibition may thus be working by inducing the clearance of these defective components. Since these clearance mechanisms seem upregulated with age, mTOR inhibition is enhancing a naturally occurring attempt at adaptation by the cells.

## Supplementary Material

Supplementary Figures

Supplementary Data 1

Supplementary Data 2

Supplementary Data 3

Supplementary Data 4

Supplementary Data 5

Supplementary Data 6

Supplementary Data 7
