# Supplementary material for: A combination of differential expression and network connectivity analyses identifies a common set of RNA splicing and processing genes altered with age across human tissues
Source: Aging (Albany NY). 2025 Dec 22;17(12):2916–49. doi: 10.18632/aging.206347 (PMC13626699; doi:10.18632/aging.206347)
Supplement: Supplementary file 1 [file aging-17-12-206347-s001.pdf]

SUPPLEMENTARY FIGURES

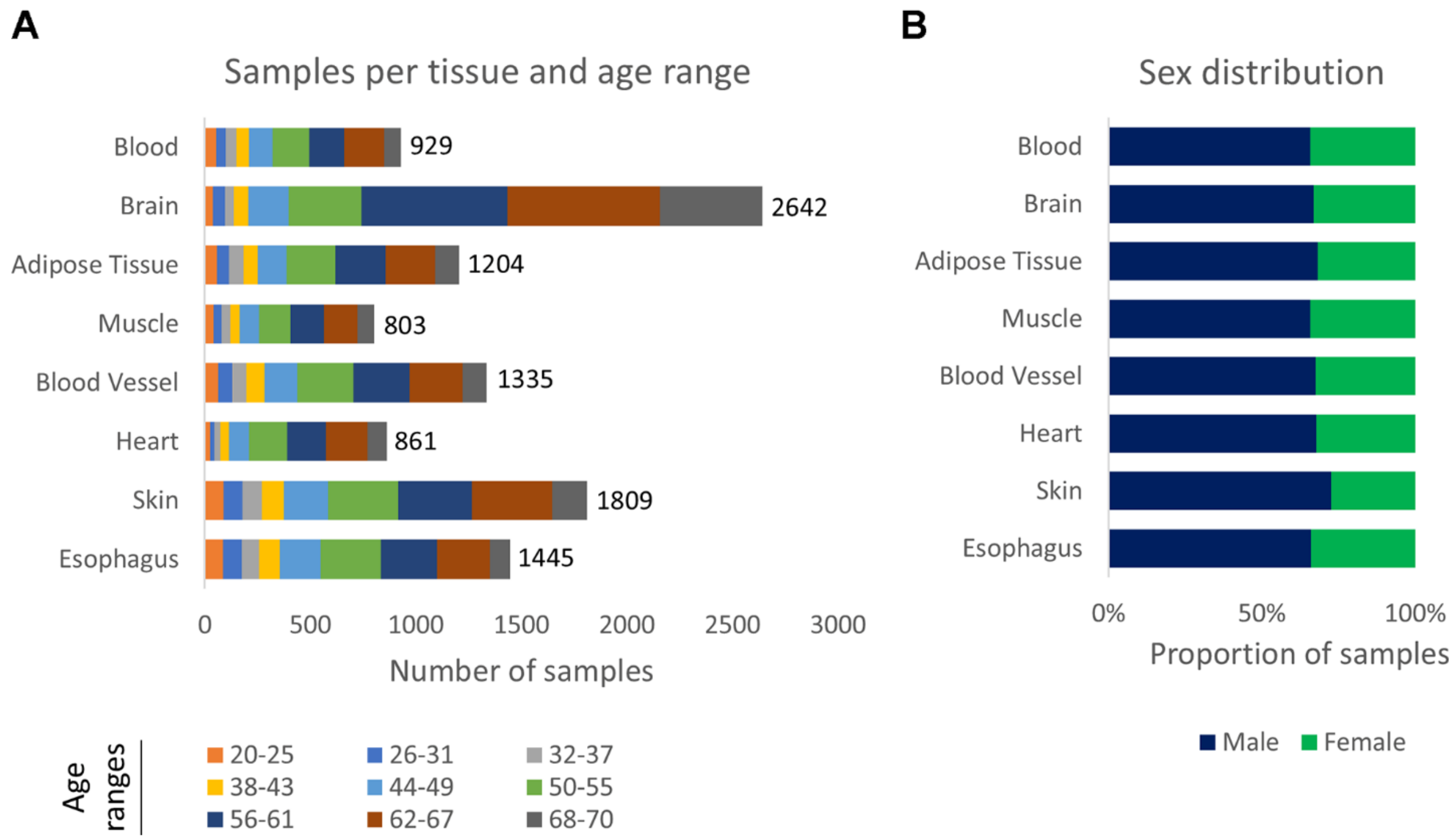

Supplementary Figure 1. Dataset description. (**A**) Number of samples per tissue per age range; age ranges (shown below) account for 6 years each, except for the last one, which accounts for 3 years. (**B**) Proportion of male and female samples in each tissue.

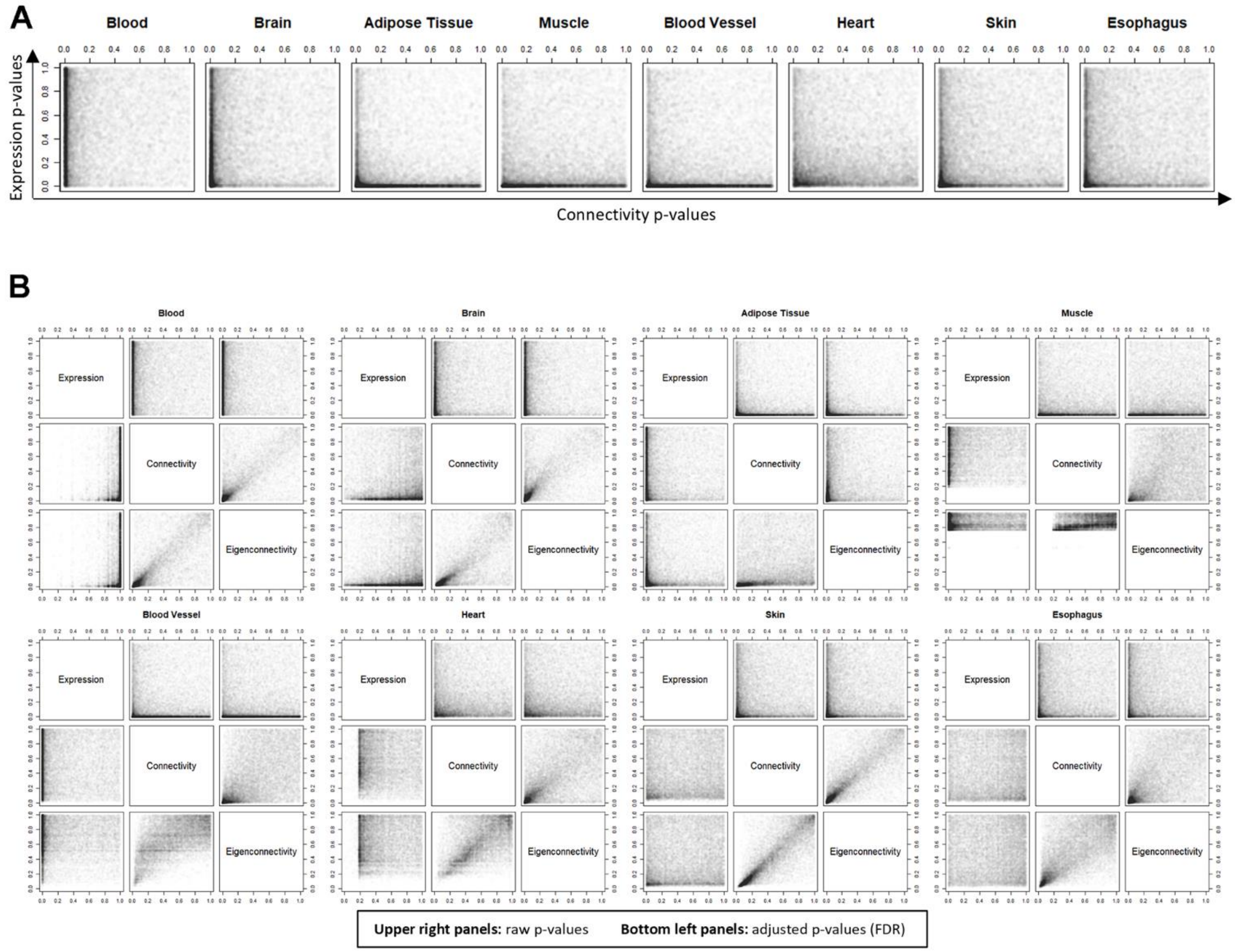

Supplementary Figure 2. Altered genes per tissue. (**A**) Distribution of raw p-values in each tissue only for differential expression and differential connectivity. (**B**) Distribution of raw p-values (upper right panels) and FDR adjusted p-values (bottom left panels) in each tissue for the three regressions.

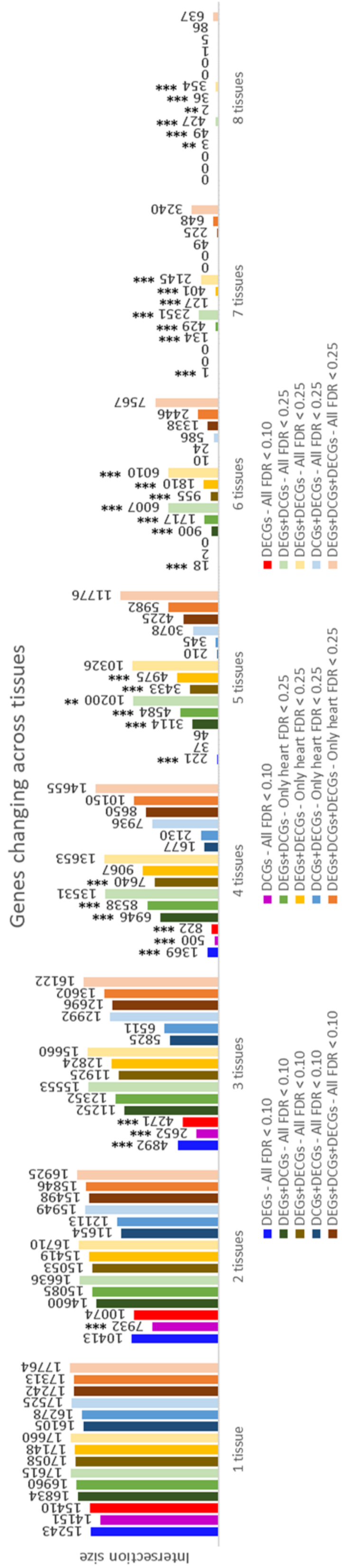

Supplementary Figure 3. Intersect counts. Number of DEGs, DCGs, DECGs, and combinations of these metrics detected in sets of 1 up to 8 tissues. The charts display the number of genes in the set and their significance in a permutation test. Significance levels: FDR<0.1 (*), FDR<0.01 (**), FDR<0.001 (***).

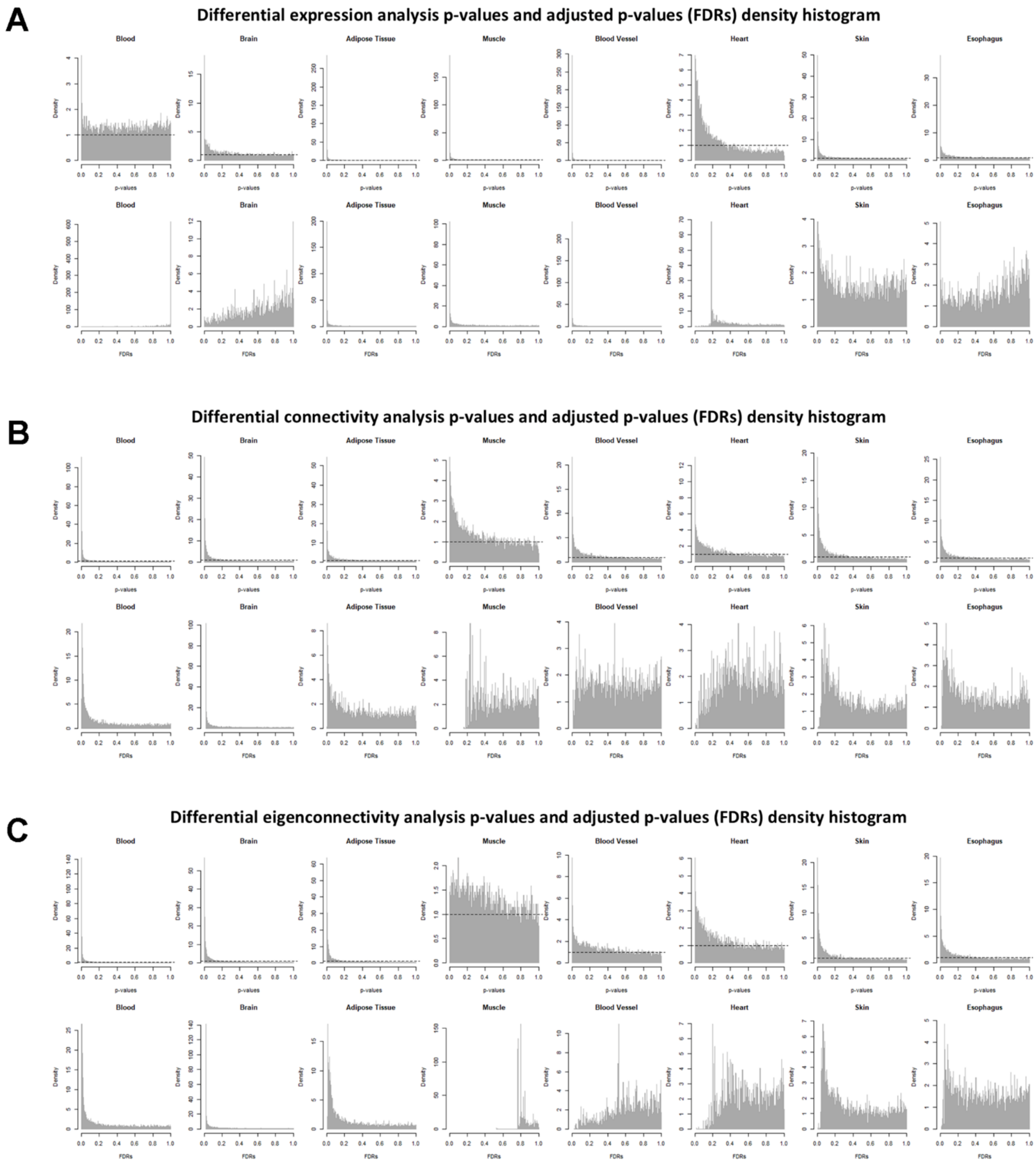

Supplementary Figure 4. Density histogram of p-values and adjusted p-values (FDRs) of the regression analyses in each tissue. (**A**) Distribution of p-values and adjusted p-values (FDRs) in the differential expression analysis. (**B**) Distribution of p-values and adjusted p-values (FDRs) in the differential connectivity analysis. (**C**) Distribution of p-values and adjusted p-values (FDRs) in the differential eigenconnectivity analysis. Dashed horizontal lines in the raw p-value density histograms represent what would be the expected distribution of p-values if the null hypothesis of no change was true for all genes.

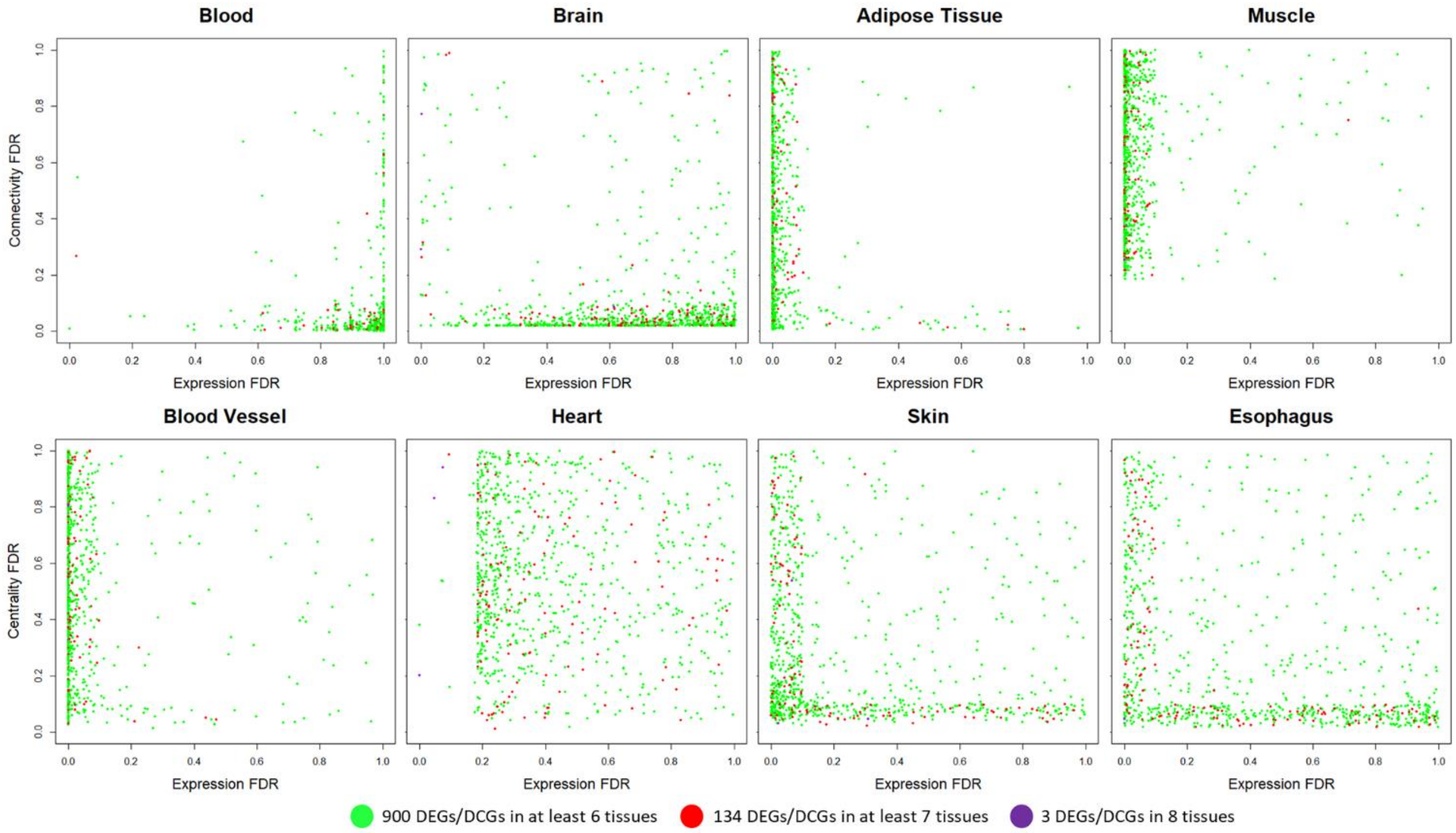

Supplementary Figure 5. Scatterplots with FDR distributions in both regressions with intersecting genes highlighted. Green points represent the 900 DEGs/DCGs in at least 6 tissues, red points represent the 134 DEGs/DCGs in at least 7 tissues, and purple points represent the 3 DEGs/DCGs in 8 tissues.

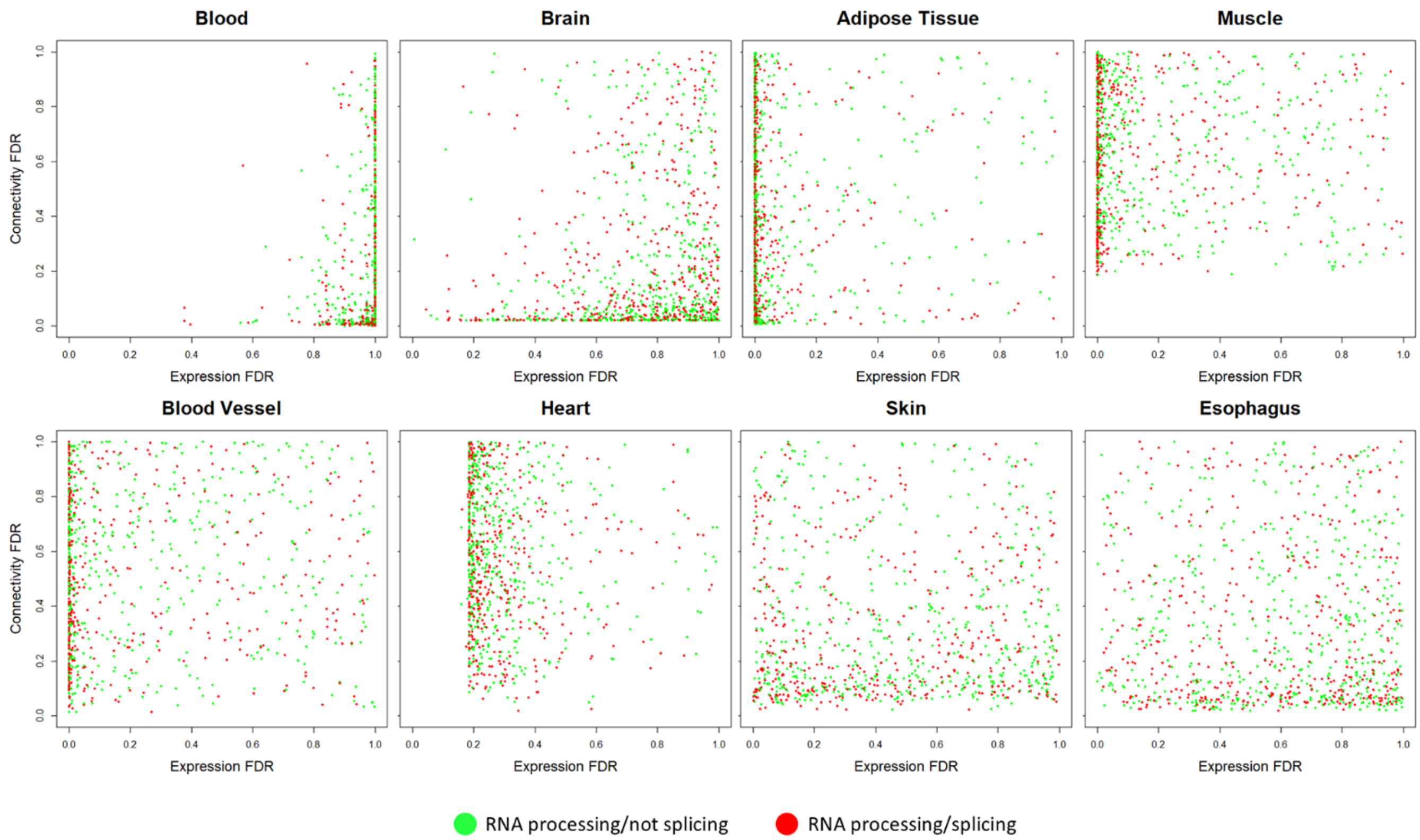

Supplementary Figure 6. Distribution of genes annotated as "RNA processing" or "RNA splicing" in the FDR distribution charts.

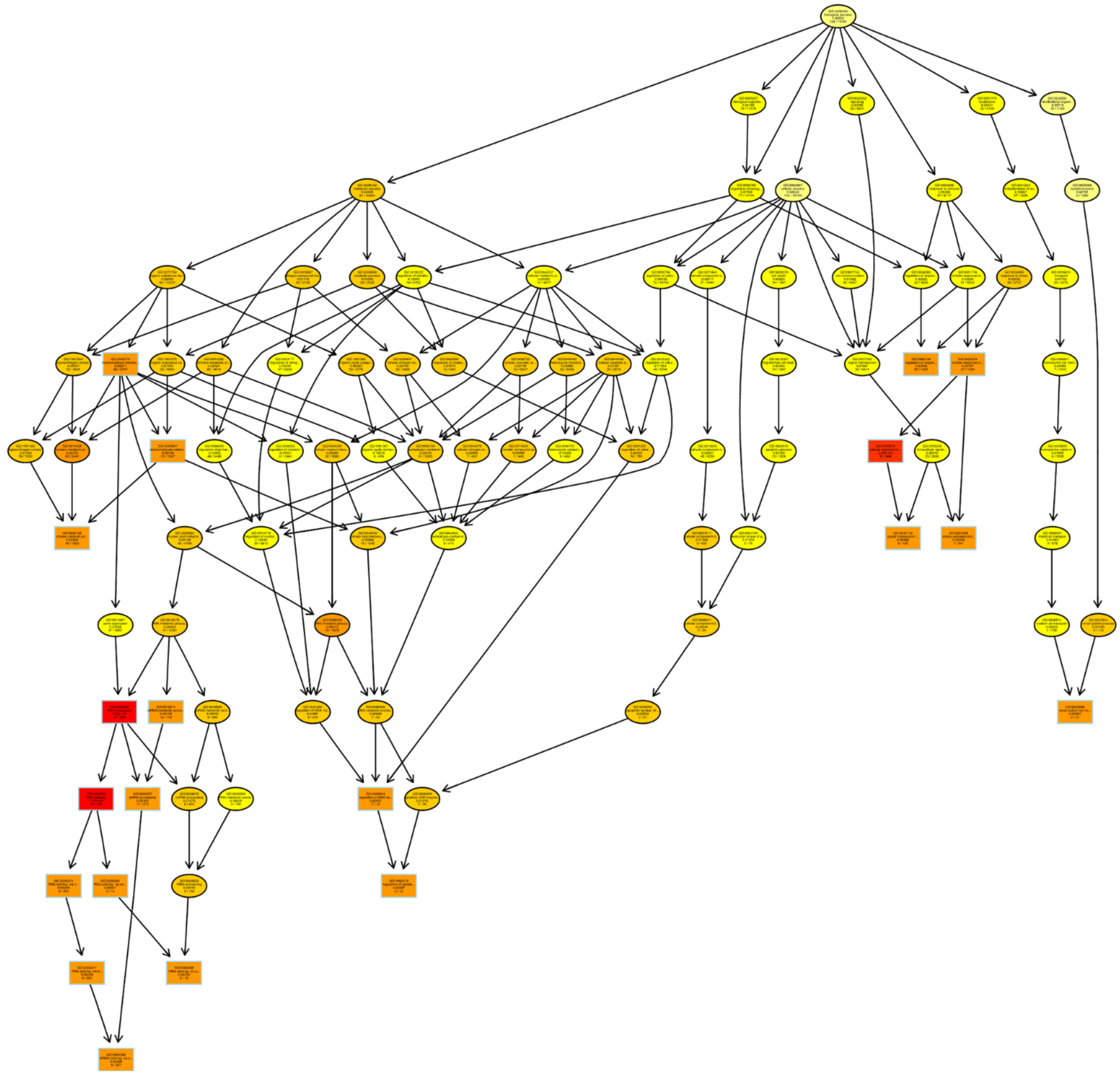

Supplementary Figure 7. Induced GO subgraph of the 20 top enriched nodes in the 134 set.

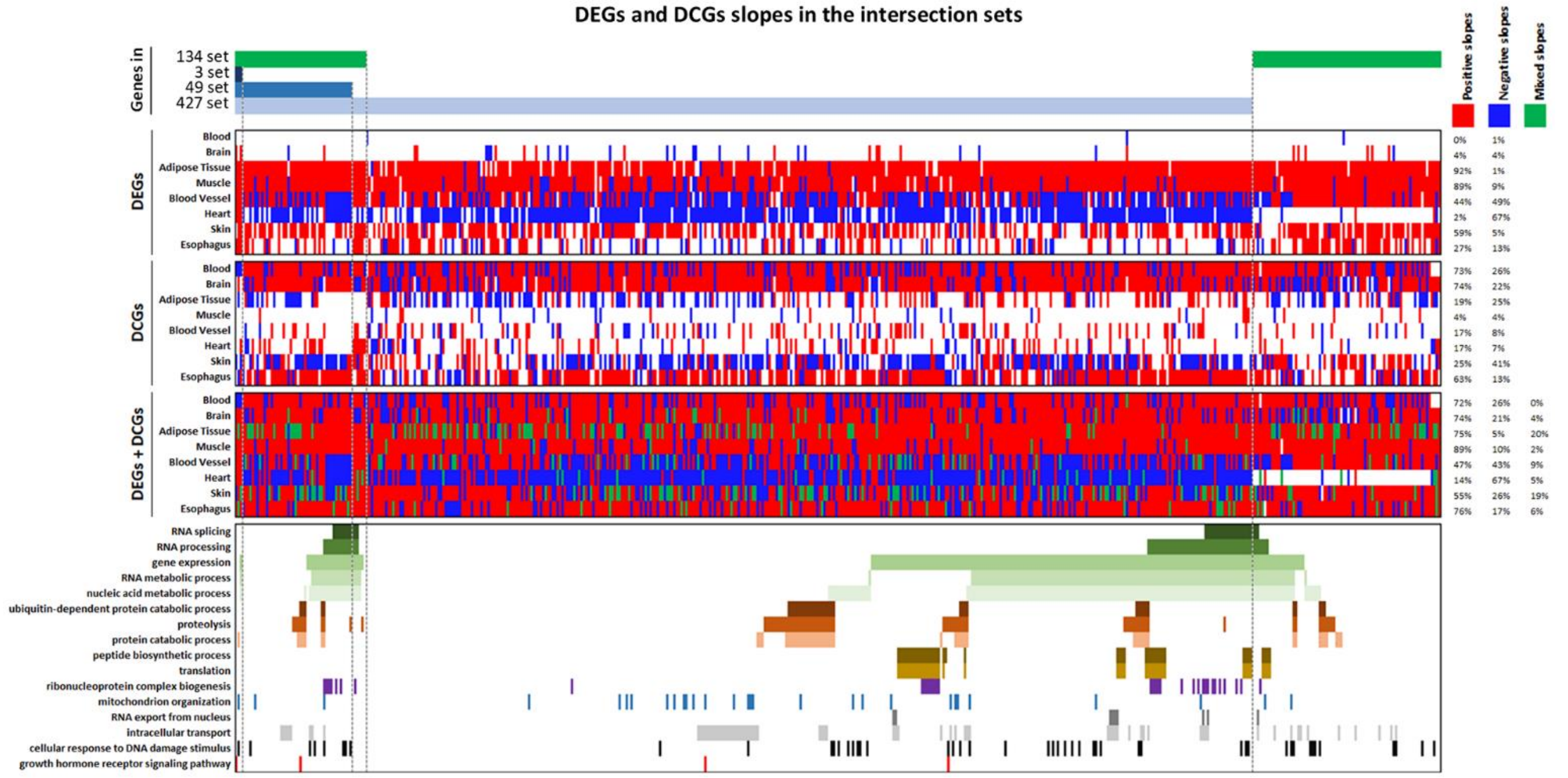

Supplementary Figure 8. Slopes of DEGs and DCGs in the selected intersection sets. In the two upper heatmaps, red entries represent positive and blue entries represent negative slopes. In the bottom heatmap, red entries represent genes that are either a positive DEG, a positive DCG, or positive in both regressions; blue entries represent genes that are either a negative DEG, a negative DCG, or negative in both regressions; and green entries indicate genes significant in both regressions, but the slopes have opposite signals (either a positive DEG and negative DCG or a negative DEG and positive DCG). The most annotated terms were selected and displayed in the last panel.

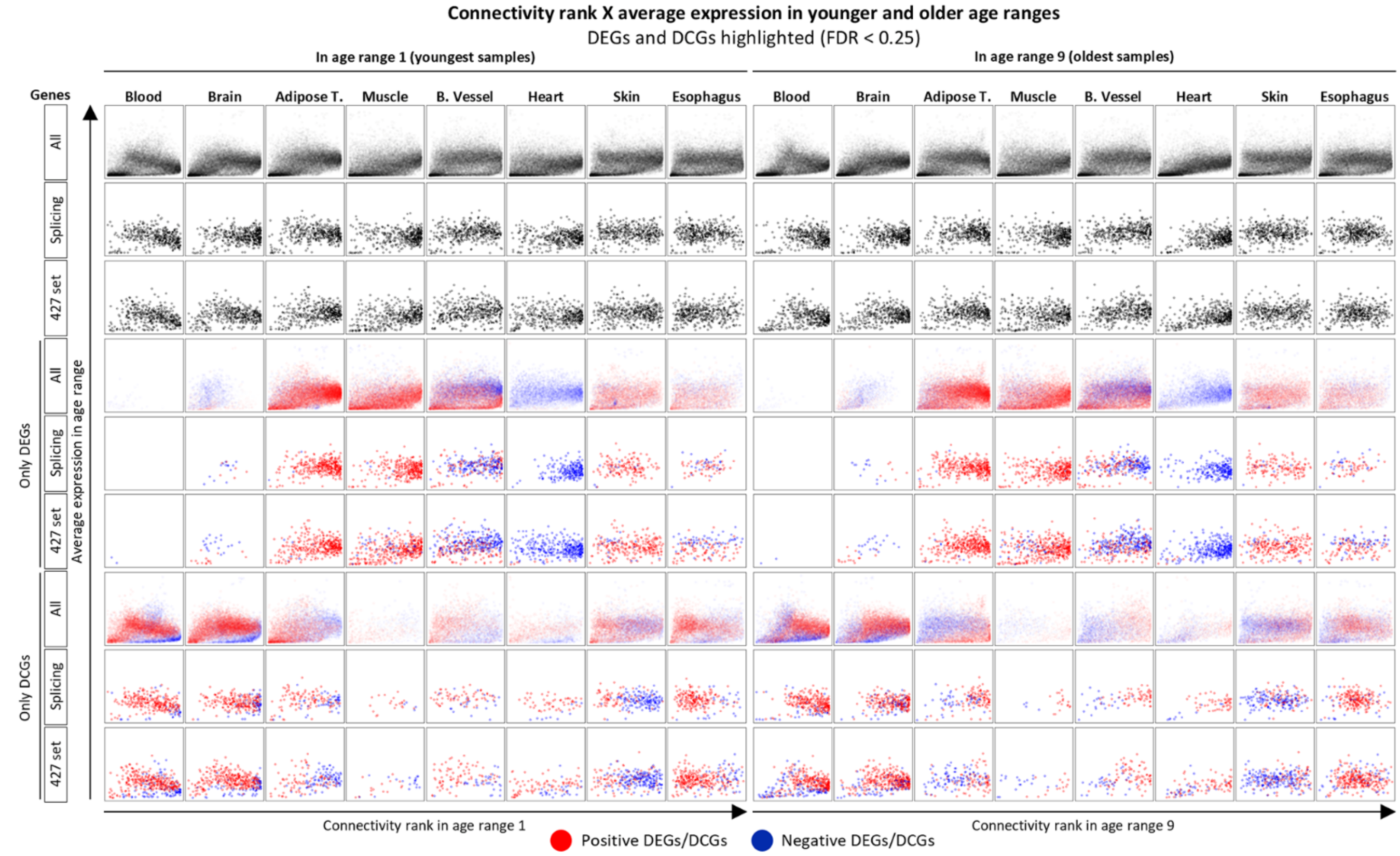

Supplementary Figure 9. Connectivity ranks x average expression of different sets of genes in age ranges 1 or 9, with DEGs and DCGs highlighted. DEGs and DCGs highlighted considered FDR < 0.25, to emphasize the overall patterns of the trajectories. Results with FDR < 0.1 can be found in Supplementary Figure 6.

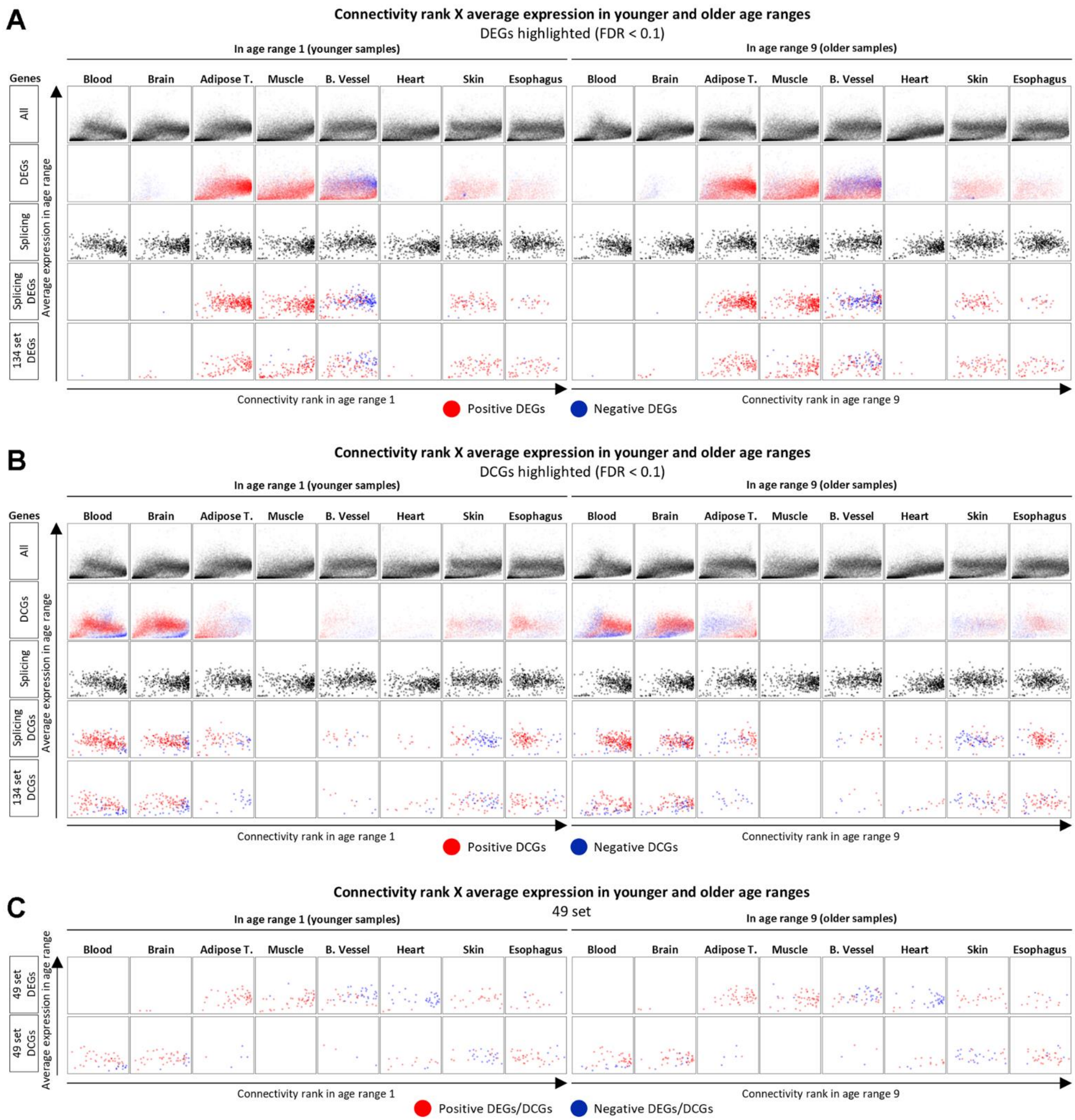

Supplementary Figure 10. Distribution of positive and negative slopes among DEGs and DCGs. (**A**) Connectivity ranks x average expression of different sets of genes in age ranges 1 or 9, with DEGs highlighted with FDR < 0.1. (**B**) Connectivity ranks x average expression of different sets of genes in age ranges 1 or 9, with DCGs highlighted with FDR < 0.1. (**C**) Connectivity ranks x average expression of the 49-set genes in age range 1 or 9, with DEGs and DCGs highlighted with FDR < 0.1 for all tissues except heart, with an FDR < 0.25. (**C**) only displays the charts for the 49-set as the charts for other tissues with FDR < 0.1 or FDR < 0.25 are shown in Figures S5, S5A and S5B.

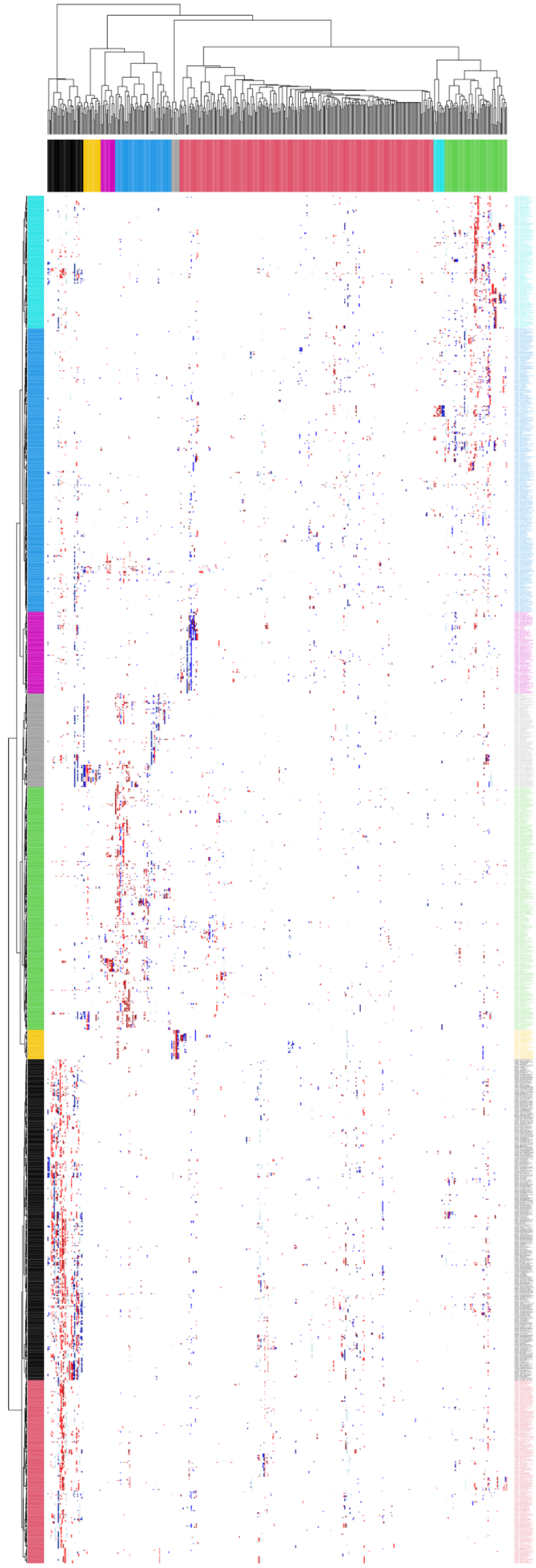

Supplementary Figure 11. Consensus Modules Enrichment Heatmap. Larger version of the heatmap in Figure 5A, displaying all labels.

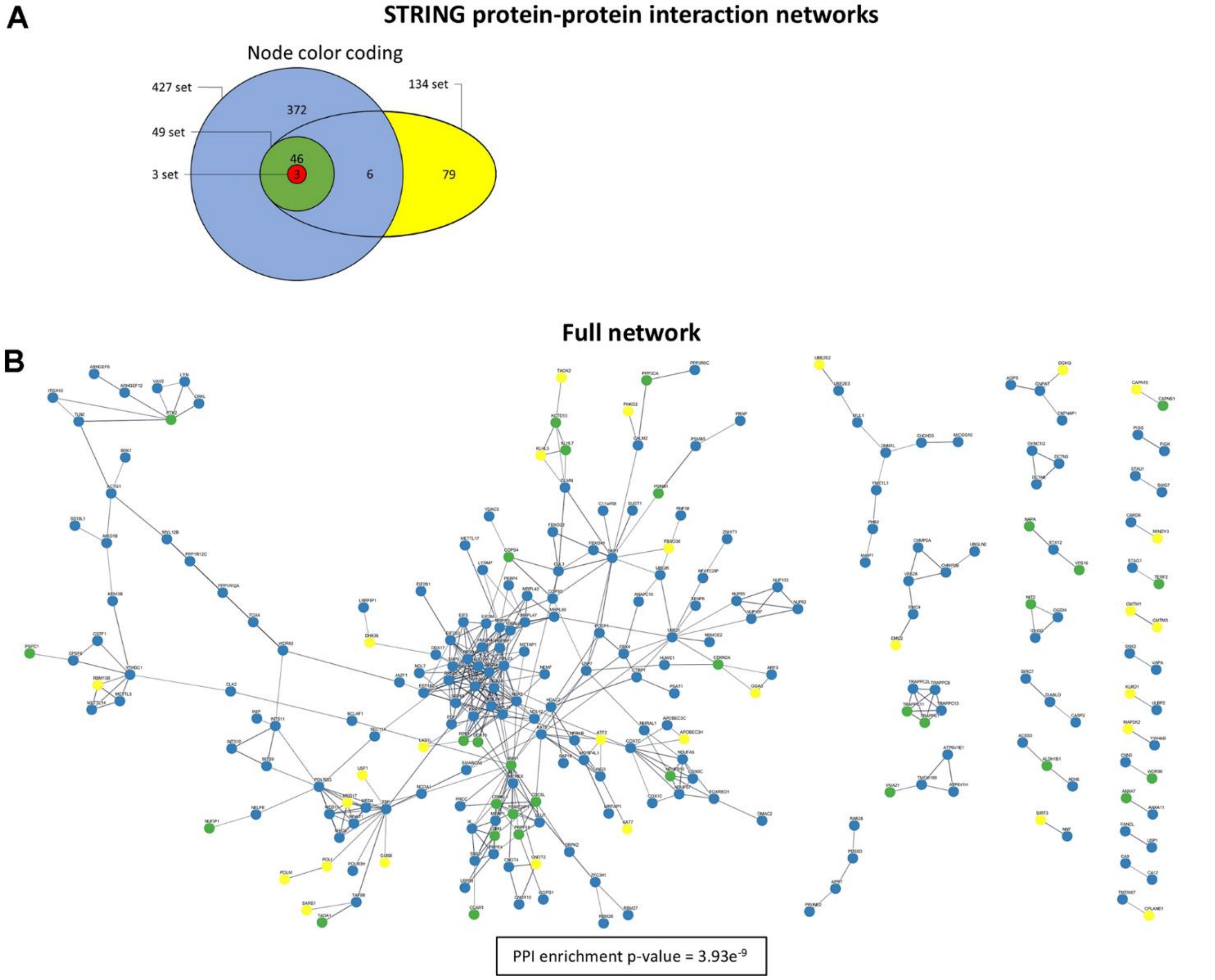

Supplementary Figure 12. STRING PPI full network. (**A**) Color coding for the nodes in the network indicating to which intersection set the corresponding genes belong. (**B**) Full interaction network, including only links with a confidence score of at least 0.7 in STRING; singletons (unconnected nodes) are omitted. The larger cluster in the middle represents ribosomal proteins, and the cluster below it, RNA splicing genes.
